# Identifying Data-Driven Clinical Subgroups for Cervical Cancer Prevention With Machine Learning: Population-Based, External, and Diagnostic Validation Study

**DOI:** 10.2196/67840

**Published:** 2025-03-19

**Authors:** Zhen Lu, Binhua Dong, Hongning Cai, Tian Tian, Junfeng Wang, Leiwen Fu, Bingyi Wang, Weijie Zhang, Shaomei Lin, Xunyuan Tuo, Juntao Wang, Tianjie Yang, Xinxin Huang, Zheng Zheng, Huifeng Xue, Shuxia Xu, Siyang Liu, Pengming Sun, Huachun Zou

**Affiliations:** 1 School of Public Health (Shenzhen), Sun Yat-sen University, Shenzhen, China; 2 Department of Gynecology, Laboratory of Gynecologic Oncology, Fujian Maternity and Child Health Hospital, College of Clinical Medicine for Obstetrics & Gynecology and Pediatrics, Fujian Medical University, Fuzhou, China; 3 Fujian Key Laboratory of Women and Children’s Critical Diseases Research, Fuzhou, China; 4 Department of Gynecology, Maternal and Child Health Hospital of Hubei Province (Women and Children's Hospital of Hubei Province) Wuhan, Wuhan, China; 5 School of Public Health, Xinjiang Medical University, Urumqi, China; 6 Division of Pharmacoepidemiology and Clinical Pharmacology, Utrecht Institute for Pharmaceutical Sciences, Utrecht University, Utrecht, Netherlands; 7 Beijing Chest Hospital, Capital Medical University, Beijing Tuberculosis and Thoracic Tumor Research Institute, Beijing, China; 8 Institute for HIV/AIDS Control and Prevention, Guangdong Provincial Center for Disease Control and Prevention, Guangzhou, China; 9 Department of HIV/AIDS Control and Prevention, Guangdong Provincial Academy of Preventive Medicine, Guangzhou, China; 10 Department of Gynecology, Shunde Women's and Children's Hospital of Guangdong Medical University, Foshan, China; 11 Department of Gynecology, Gansu Provincial Maternity and Child-care Hospital, Lanzhou, China; 12 Department of Gynecology, Guiyang Maternal and Child Health Care Hospital, Guiyang, China; 13 Department of Gynecology, Shenzhen Maternity & Child Healthcare Hospital, Shenzhen, China; 14 The Ministry of Health, Fujian Maternity and Child Health Hospital, College of Clinical Medicine for Obstetrics & Gynecology and Pediatrics, Fujian Medical University, Fuzhou, China; 15 Center for Cervical Disease Diagnosis and Treatment, Fujian Maternity and Child Health Hospital, College of Clinical Medicine for Obstetrics & Gynecology and Pediatrics, Fujian Medical University, Fuzhou, China; 16 Department of Pathology, Fujian Maternity and Child Health Hospital, College of Clinical Medicine for Obstetrics & Gynecology and Pediatrics, Fujian Medical University, Fuzhou, China; 17 School of Group Medicine and Public Health, Peking Union Medical College, Beijing, China; 18 School of Public Health, Fudan University, Shanghai, China; 19 Shenzhen Campus, Sun Yat-sen University, Shenzhen, China; 20 Fujian Maternity and Child Health Hospital, College of Clinical Medicine for Obstetrics and Gynecology and Pediatrics, Fujian Medical University, Fuzhou, China

**Keywords:** cervical cancer, human papillomavirus, screening, machine learning, cervical tumor, cancer, carcinoma, tumor, malignant, ML, phenomapping strategy, logistic regression, regression, population-based, validation study, cancer prevention, validity, usability, algorithm, surveillance, electronic health record, EHR

## Abstract

**Background:**

Cervical cancer remains a major global health issue. Personalized, data-driven cervical cancer prevention (CCP) strategies tailored to phenotypic profiles may improve prevention and reduce disease burden.

**Objective:**

This study aimed to identify subgroups with differential cervical precancer or cancer risks using machine learning, validate subgroup predictions across datasets, and propose a computational phenomapping strategy to enhance global CCP efforts.

**Methods:**

We explored the data-driven CCP subgroups by applying unsupervised machine learning to a deeply phenotyped, population-based discovery cohort. We extracted CCP-specific risks of cervical intraepithelial neoplasia (CIN) and cervical cancer through weighted logistic regression analyses providing odds ratio (OR) estimates and 95% CIs. We trained a supervised machine learning model and developed pathways to classify individuals before evaluating its diagnostic validity and usability on an external cohort.

**Results:**

This study included 551,934 women (median age, 49 years) in the discovery cohort and 47,130 women (median age, 37 years) in the external cohort. Phenotyping identified 5 CCP subgroups, with CCP4 showing the highest carcinoma prevalence. CCP2–4 had significantly higher risks of CIN2+ (CCP2: OR 2.07 [95% CI: 2.03‐2.12], CCP3: 3.88 [3.78‐3.97], and CCP4: 4.47 [4.33‐4.63]) and CIN3+ (CCP2: 2.10 [2.05‐2.14], CCP3: 3.92 [3.82‐4.02], and CCP4: 4.45 [4.31‐4.61]) compared to CCP1 (*P*<.001), consistent with the direction of results observed in the external cohort. The proposed triple strategy was validated as clinically relevant, prioritizing high-risk subgroups (CCP3-4) for colposcopies and scaling human papillomavirus screening for CCP1-2.

**Conclusions:**

This study underscores the potential of leveraging machine learning algorithms and large-scale routine electronic health records to enhance CCP strategies. By identifying key determinants of CIN2+/CIN3+ risk and classifying 5 distinct subgroups, our study provides a robust, data-driven foundation for the proposed triple strategy. This approach prioritizes tailored prevention efforts for subgroups with varying risks, offering a novel and scalable tool to complement existing cervical cancer screening guidelines. Future work should focus on independent external and prospective validation to maximize the global impact of this strategy.

## Introduction

Cervical cancer is the fourth most common cancer among women, with an estimated 660,000 new cases and 350,000 deaths globally in 2022 [1,2]. It is the most common cancer in 25 countries and the leading cause of cancer death in 37 countries. Despite being largely preventable through human papillomavirus (HPV) vaccination [3], the high incidence and persistence of high-risk HPV (hrHPV) remain the primary risk factors for cervical cancer [4] and related diseases [5-7]. In November 2020, the World Health Organization (WHO) launched a global initiative [8] to eliminate cervical cancer as a public health problem, emphasizing a triple intervention strategy: vaccinating at least 90% of girls against HPV by age 15 years, screening 70% of women with a high-performance test by ages 35 and 45 years, and treating at least 90% of detected precancerous lesions and invasive cancers. Yet, globally, an estimated 1.6 billion (67%) of 2.3 billion women aged 20‐70 years have never been screened for cervical cancer [9], and in China, 5-year screening coverage among women aged 35‐49 years was only 33%. Establishing high-quality, sustainable, and acceptable cervical cancer prevention (CCP) with broad coverage in resource-limited regions remains a critical challenge [10-12]. While HPV-based screening has demonstrated significant benefits [13-18], leading the WHO to recommend it as the primary method for CCP globally [19], its adoption in low- and middle-income countries (LMICs) is limited due to health inequities, resource constraints, and limited access to affordable, clinically validated HPV tests [9,20]. Addressing these barriers requires personalized CCP strategies.

We hypothesize that data-driven CCP subgroups, identified using machine learning, can provide valuable insights into CCP, enabling personalized strategies to reduce the risk of cervical intraepithelial neoplasia (CIN) and cervical cancer. Personalized CCP strategies depend on the unique phenotypic profile of each individual. The population, with its varying phenotypic diversity, comprises heterogeneous subgroups that reflect multiple underlying behavior patterns and causes of disease. Clinical practice guidelines also recommend that cervical cancer screening should be tailored to an individual’s risk profile of HPV infection [21,22]. Therefore, the discovery of CCP subgroups could inform and improve the development of new screening strategies, public health policies, and clinical decision-making, as well as contribute to trial design, despite the complex causal relationships among individual risk factors [4]. To date, no study has identified CCP subgroups. The incomplete understanding of these subgroups across large, diverse populations, coupled with insufficient validation through various methods, has hindered the implementation of the triple intervention strategy. Machine learning-based approaches allow for the phenotyping of entire populations based on individual characteristics, enabling the prediction of phenotypic clusters and disease onset [23,24]. Additionally, by defining a computational phenomap—a mathematical construct of individual phenotypes based on baseline measures [24]—computational methods can assess the heterogeneous risk effects of distinct subpopulations. This approach accounts for the phenotypic diversity within populations and their subsequent histopathological diagnoses.

As such, in this study, we aimed to: (1) test the hypothesis that distinct CCP subgroups exhibit differential risks of cervical precancer or cancer based on their complex phenotypic profiles (development); (2) demonstrate internal validity (within a dataset and across methods), as well as external and diagnostic validity (across datasets; validation); and (3) propose a computational phenomapping strategy with clinical relevance and pathways to improve global access to CCP (impact).

## Methods

### Data Source

**Figure 1.**
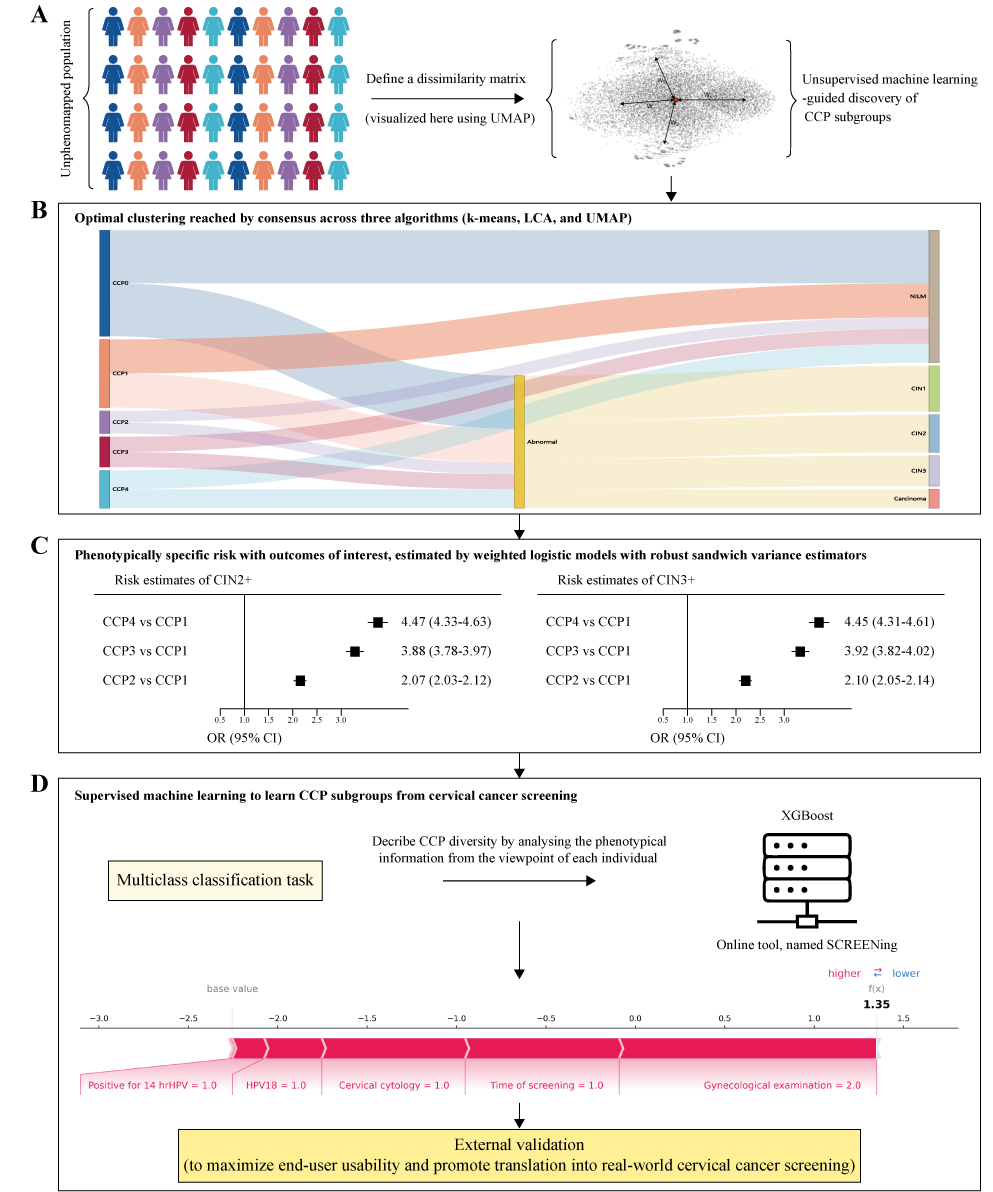
Study design. (**A**) We explored the data-driven cervical cancer prevention (CCP) subgroups by applying unsupervised machine learning to a deeply phenotyped, population-based discovery cohort. In this population-based, external, and diagnostic validation study, we aimed to use different machine learning methods to test our hypothesis (**B**) that individuals within distinct CCP subgroups exhibit differential risks of cervical precancer or cancer based on their complex phenotypic profiles. After identifying CCP subgroups, we extracted CCP-specific risks of outcomes of interest (**C**) conditionally on all predefined and algorithmically selected features through weighted logistic regression analyses providing average odds ratio (OR) estimates. Additionally, we stratified individuals based on their key features to conduct subgroup analyses. Finally, we trained a supervised machine learning model and developed pathways (**D**) to classify individuals using features most consistently linked to CCP subgroups before evaluating its diagnostic validity and usability on an external cohort. Pathologically abnormal diagnoses consisted of CIN1, CIN2, CIN3, and carcinoma. UMAP: uniform manifold approximation and projection; CCP: cervical cancer prevention; LCA: latent class analysis; NILM: negative for intraepithelial lesion or malignancy; CIN1: cervical intraepithelial neoplasia grade 1; CIN2: cervical intraepithelial neoplasia grade 2; CIN3: cervical intraepithelial neoplasia grade 3; CIN2+: cervical intraepithelial neoplasia grade 2 or worse; CIN3+: cervical intraepithelial neoplasia grade 3 or worse; OR: odds ratio; XGBoost: eXtreme gradient boosting; SCREENing: clinical Subgroups for CeRvical cancEr prEvention using computational phenomaps and machine learning; hrHPV: high-risk human papillomavirus; HPV: human papillomavirus.

Deidentified data were extracted from electronic health records (EHRs) of the national cervical cancer screening program in China. In summary, our study included eligible women aged 25‐65 years who participated in the cervical cancer screening. Data from Fujian Province (2014‐2023) were used to establish a discovery cohort to train the models. Additionally, EHR data from 5 other regions—Shenzhen City, Foshan City, Hubei Province, Gansu Province, and Guizhou Province—were employed as an external cohort to validate the generalizability of the models across diverse populations. Details on the study design, as illustrated in Figure 1, are available in Appendix S1 in Multimedia Appendix 1.

### Ethical Considerations

This study was approved by the Ethics Committee of Fujian Maternal and Child Health Hospital (2023KY141). No additional informed consent was required for this analysis. All participant data were deidentified prior to analysis. No images of identifiable individuals are included in the manuscript or supplementary materials.

### Data Preprocessing

In line with the consensus that detecting and treating CIN2 or CIN3 [25,26], key premalignant cervical conditions, can prevent progression to invasive cervical cancer, our primary and secondary outcomes were CIN2+ and CIN3+, respectively. These outcomes were confirmed by histopathology and align with those commonly used in previous studies [26-29]. We selected features based on prior research [22,30-34] and input from clinical, biostatistical, and epidemiological experts. These included demographic characteristics, cervical cancer screening history, HPV infection status, and medical examination results. Details on data preprocessing are provided in Appendix S1 in Multimedia Appendix 1.

### Unsupervised Machine Learning

We calculated phenotypic distances between individuals using Gower’s distance [35], a dissimilarity metric suitable for mixed continuous and categorical data. To visualize phenotypic variation in the population, we employed uniform manifold approximation and projection (UMAP) [36] to construct a phenomap. This approach enhances interpretability by presenting the distributions of individuals within a multidimensional phenotypic space, capturing the full range of baseline phenotypes. To identify and categorize the underlying CCP subgroups in which individuals exhibit phenotypic similarity, we employed and compared 3 methods to capture and validate CCP diversity: k-means [37], latent class analysis [38], and UMAP [39]. The final number of CCP subgroups was determined by reaching a consensus across all 3 approaches. Further details are provided in Appendix S1 in Multimedia Appendix 1.

### Risk Estimates and an Algorithm to Identify Subgroups

To further explore the association between CCP subgroups and outcomes of interest, we calculated CCP-specific risk estimates (odds ratios, ORs) [40] and 95% CIs using inverse probability weights [41]. Additionally, we trained an eXtreme gradient boosting (XGBoost) algorithm [42] to predict 5 CCP subgroups with differential risks of outcomes, as described in our previous work [23]. Briefly, the model was trained and calibrated using an isotonic regression algorithm, and internally validated in the discovery cohort. The SHapley Additive exPlanations (SHAP) method was employed to identify each feature’s relative contribution [23,43] and enhance the model’s explainability. Model performance was evaluated using OVR AUROC (one-versus-rest area under the receiver operating characteristics curve, extended for multiple classes), Brier score, and calibration curves as primary metrics. In external validation, the model’s diagnostic validity was assessed by comparing CCP-specific ORs between datasets to evaluate cross-cluster and cross-dataset risk differences. Further details are provided in Appendix S1 in Multimedia Appendix 1.

### Developing Pathways to Improve Impact

We named our computational phenomapping strategy SCREENing (clinical Subgroups for CeRvical cancEr prEvention using computational pheNomaps and machine learnING). To assess real-world usability and effectiveness, we consulted 11 clinical experts and 3 epidemiologists on its clinical relevance, justification, result interpretability, and potential impact on screening strategies and public health policymaking.

### Statistical Analysis

Given the importance of menopause in women, we specifically examined CCP-specific risks across age using spline analyses, with interaction tests to assess whether age modified these risks. Subgroup analyses were also performed by stratifying women based on key features. Details are provided in Appendix S1 in Multimedia Appendix 1. We followed the Guidelines for Developing and Reporting Machine Learning Predictive Models in Biomedical Research [44] and the Transparent Reporting of a multivariable prediction model for Individual Prognosis or Diagnosis statement [45]. Data management was performed using the lulab.utils R package [46]. All analyses [47] were conducted using Python (version 3.11.6), SAS Enterprise Guide (version 7.1), and R (version 4.3.2; R Foundation for Statistical Computing).

## Results

### Characteristics of Cohorts

The study included 551,934 women (median age 49 years [42,48]; 10% infected with hrHPV, 1.2% with HPV-16, and 0.6% with HPV-18) in the discovery cohort (Table 1) and 47,130 women (median age 37 years [32,45]; 16.6% with hrHPV, 3.9% with HPV-16, and 1.4% with HPV-18) in the external cohort (Table S3 in Multimedia Appendix 1). In the discovery cohort, 9932 were pathologically diagnosed with cervical abnormalities, including 533 with carcinoma. In contrast, the external cohort showed a higher prevalence of cervical abnormalities at 11.1% (5251).

**Table 1.** Characteristics by cervical cancer prevention (CCP) subgroups in the discovery cohort. Categorical features are summarized as numbers (percentages), and continuous features as median (Q1, Q3), as appropriate.

Characteristics	CCP0^a^ (n=542,002)	CCP1 (n=2242)	CCP2 (n=3770)	CCP3 (n=2278)	CCP4 (n=1642)	Total (n=551,934)
Age (years), median (Q1, Q3)	48.00 (42.00, 54.00)	48.00 (42.00, 54.00)	48.00 (43.00, 55.00)	51.00 (44.00, 57.00)	48.00 (43.00, 54.00)	49.00 (42.00, 54.00)
**Race/ethnicity, n (%)**						
Han	534,023 (98.53)	2214 (98.75)	3707 (98.33)	2236 (98.16)	1607 (97.87)	543,787 (98.52)
Others	4528 (0.84)	15 (0.67)	30 (0.80)	29 (1.27)	20 (1.22)	4622 (0.84)
Missing	3451 (0.64)	13 (0.58)	33 (0.88)	13 (0.57)	15 (0.91)	3525 (0.64)
**History of cervical cancer screening, n (%)**						
Missing	234 (0.04)	0 (0.00)	2 (0.05)	0 (0.00)	0 (0.00)	236 (0.04)
No	411,029 (75.84)	0 (0.00)	3768 (99.95)	2278 (100.00)	1642 (100.00)	418,717 (75.86)
Yes	130,739 (24.12)	2242 (100.00)	0 (0.00)	0 (0.00)	0 (0.00)	132,981 (24.09)
**Time of previous screening, n (%)**						
Missing	785 (0.14)	13 (0.58)	2 (0.05)	0 (0.00)	0 (0.00)	800 (0.14)
No previous screening	411,029 (75.84)	0 (0.00)	3768 (99.95)	2278 (100.00)	1642 (100.00)	418,717 (75.86)
Within 3 years from now	65,519 (12.09)	1091 (48.66)	0 (0.00)	0 (0.00)	0 (0.00)	66,610 (12.07)
More than 3 years ago	64,669 (11.93)	1138 (50.76)	0 (0.00)	0 (0.00)	0 (0.00)	65,807 (11.92)
**Gynecological examination, n (%)**						
Missing	2180 (0.40)	1 (0.04)	1 (0.03)	0 (0.00)	0 (0.00)	2182 (0.40)
Normal	417,921 (77.11)	1508 (67.26)	3763 (99.81)	1568 (68.83)	0 (0.00)	424,760 (76.96)
Abnormal	121,901 (22.49)	733 (32.69)	6 (0.16)	710 (31.17)	1642 (100.00)	124,992 (22.65)
**Positive for high-risk HPV** b c **, n (%)**						
No	496,234 (91.56)	99 (4.42)	206 (5.46)	27 (1.19)	67 (4.08)	496,633 (89.98)
Yes	45,768 (8.44)	2143 (95.58)	3564 (94.54)	2251 (98.81)	1575 (95.92)	55,301 (10.02)
**Positive for low-risk HPV** ^d^ **, n (%)**						
No	534,313 (98.58)	2035 (90.77)	3664 (97.19)	1813 (79.59)	1602 (97.56)	543,427 (98.46)
Yes	7689 (1.42)	207 (9.23)	106 (2.81)	465 (20.41)	40 (2.44)	8507 (1.54)
**Positive for possible high-risk HPV** ^e^ **, n (%)**						
No	537,999 (99.26)	2130 (95.00)	3686 (97.77)	1987 (87.23)	1617 (98.48)	547,419 (99.18)
Yes	4003 (0.74)	112 (5.00)	84 (2.23)	291 (12.77)	25 (1.52)	4515 (0.82)
Number of HPV infections	0.00 (0.00, 0.00)	1.00 (1.00, 2.00)	1.00 (1.00, 1.00)	2.00 (2.00, 3.00)	1.00 (1.00, 1.00)	0.00 (0.00, 0.00)
**Positive for HPV-16, n (%)**						
No	538,375 (99.33)	1684 (75.11)	2745 (72.81)	1494 (65.58)	1199 (73.02)	545,497 (98.83)
Yes	3627 (0.67)	558 (24.89)	1025 (27.19)	784 (34.42)	443 (26.98)	6437 (1.17)
**Positive for HPV-18, n (%)**						
No	539,716 (99.58)	2002 (89.30)	3442 (91.30)	1887 (82.84)	1508 (91.84)	548,555 (99.39)
Yes	2286 (0.42)	240 (10.70)	328 (8.70)	391 (17.16)	134 (8.16)	3379 (0.61)
**Positive for HPV-31, n (%)**						
No	540,143 (99.66)	2138 (95.36)	3662 (97.14)	2063 (90.56)	1588 (96.71)	549,594 (99.58)
Yes	1859 (0.34)	104 (4.64)	108 (2.86)	215 (9.44)	54 (3.29)	2340 (0.42)
**Positive for HPV-33, n (%)**						
No	539,290 (99.50)	2096 (93.49)	3608 (95.70)	1978 (86.83)	1562 (95.13)	548,534 (99.38)
Yes	2712 (0.50)	146 (6.51)	162 (4.30)	300 (13.17)	80 (4.87)	3400 (0.62)
**Positive for HPV-35, n (%)**						
No	540,820 (99.78)	2197 (97.99)	3708 (98.36)	2160 (94.82)	1622 (98.78)	550,507 (99.74)
Yes	1182 (0.22)	45 (2.01)	62 (1.64)	118 (5.18)	20 (1.22)	1427 (0.26)
**Positive for HPV-39, n (%)**						
No	537,675 (99.20)	2092 (93.31)	3638 (96.50)	2021 (88.72)	1600 (97.44)	547,026 (99.11)
Yes	4327 (0.80)	150 (6.69)	132 (3.50)	257 (11.28)	42 (2.56)	4908 (0.89)
**Positive for HPV-45, n (%)**						
No	540,998 (99.81)	2205 (98.35)	3741 (99.23)	2204 (96.75)	1632 (99.39)	550,780 (99.79)
Yes	1004 (0.19)	37 (1.65)	29 (0.77)	74 (3.25)	10 (0.61)	1154 (0.21)
**Positive for HPV-51, n (%)**						
No	537,654 (99.20)	2053 (91.57)	3590 (95.23)	1954 (85.78)	1565 (95.31)	546,816 (99.07)
Yes	4348 (0.80)	189 (8.43)	180 (4.77)	324 (14.22)	77 (4.69)	5118 (0.93)
**Positive for HPV-52, n (%)**						
No	525,904 (97.03)	1622 (72.35)	3019 (80.08)	1396 (61.28)	1276 (77.71)	533,217 (96.61)
Yes	16,098 (2.97)	620 (27.65)	751 (19.92)	882 (38.72)	366 (22.29)	18,717 (3.39)
**Positive for HPV-56, n (%)**						
No	539,439 (99.53)	2133 (95.14)	3682 (97.67)	2076 (91.13)	1610 (98.05)	548,940 (99.46)
Yes	2563 (0.47)	109 (4.86)	88 (2.33)	202 (8.87)	32 (1.95)	2994 (0.54)
**Positive for HPV-58, n (%)**						
No	534,764 (98.66)	1877 (83.72)	3297 (87.45)	1691 (74.23)	1413 (86.05)	543,042 (98.39)
Yes	7238 (1.34)	365 (16.28)	473 (12.55)	587 (25.77)	229 (13.95)	8892 (1.61)
**Positive for HPV-59, n (%)**						
No	540,034 (99.64)	2180 (97.23)	3710 (98.41)	2124 (93.24)	1620 (98.66)	549,668 (99.59)
Yes	1968 (0.36)	62 (2.77)	60 (1.59)	154 (6.76)	22 (1.34)	2266 (0.41)
**Positive for HPV-66, n (%)**						
No	540,640 (99.75)	2186 (97.50)	3720 (98.67)	2180 (95.70)	1614 (98.29)	550,340 (99.71)
Yes	1362 (0.25)	56 (2.50)	50 (1.33)	98 (4.30)	28 (1.71)	1594 (0.29)
**Positive for HPV-68, n (%)**						
No	538,250 (99.31)	2124 (94.74)	3654 (96.92)	2018 (88.59)	1604 (97.69)	547,650 (99.22)
Yes	3752 (0.69)	118 (5.26)	116 (3.08)	260 (11.41)	38 (2.31)	4284 (0.78)
**Positive for HPV-11, n (%)**						
No	541,435 (99.90)	2218 (98.93)	3764 (99.84)	2235 (98.11)	1631 (99.33)	551,283 (99.88)
Yes	567 (0.10)	24 (1.07)	6 (0.16)	43 (1.89)	11 (0.67)	651 (0.12)
**Positive for HPV-42, n (%)**						
No	540,587 (99.74)	2199 (98.08)	3745 (99.34)	2175 (95.48)	1639 (99.82)	550,345 (99.71)
Yes	1415 (0.26)	43 (1.92)	25 (0.66)	103 (4.52)	3 (0.18)	1589 (0.29)
**Positive for HPV-43, n (%)**						
No	541,315 (99.87)	2212 (98.66)	3759 (99.71)	2210 (97.01)	1641 (99.94)	551,137 (99.86)
Yes	687 (0.13)	30 (1.34)	11 (0.29)	68 (2.99)	1 (0.06)	797 (0.14)
**Positive for HPV-44, n (%)**						
No	540,505 (99.72)	2212 (98.66)	3753 (99.55)	2201 (96.62)	1636 (99.63)	550,307 (99.71)
Yes	1497 (0.28)	30 (1.34)	17 (0.45)	77 (3.38)	6 (0.37)	1627 (0.29)
**Positive for HPV-6, n (%)**						
No	540,748 (99.77)	2207 (98.44)	3748 (99.42)	2199 (96.53)	1635 (99.57)	550,537 (99.75)
Yes	1254 (0.23)	35 (1.56)	22 (0.58)	79 (3.47)	7 (0.43)	1397 (0.25)
**Positive for HPV-81, n (%)**						
No	539,354 (99.51)	2178 (97.15)	3747 (99.39)	2136 (93.77)	1632 (99.39)	549,047 (99.48)
Yes	2648 (0.49)	64 (2.85)	23 (0.61)	142 (6.23)	10 (0.61)	2887 (0.52)
**Positive for HPV-53, n (%)**						
No	538,445 (99.34)	2139 (95.41)	3689 (97.85)	2015 (88.45)	1617 (98.48)	547,905 (99.27)
Yes	3557 (0.66)	103 (4.59)	81 (2.15)	263 (11.55)	25 (1.52)	4029 (0.73)
**Cervical cytology examination, n (%)**						
NILM^f^	42,746 (7.89)	489 (21.81)	721 (19.12)	438 (19.23)	345 (21.01)	44,739 (8.11)
No examination due to negative for high-risk HPV	487,235 (89.90)	3 (0.13)	7 (0.19)	0 (0.00)	0 (0.00)	487,245 (88.28)
ASC-US^g^	7577 (1.40)	808 (36.04)	1185 (31.43)	721 (31.65)	531 (32.34)	10822 (1.96)
LSIL^h^	2245 (0.41)	488 (21.77)	772 (20.48)	546 (23.97)	286 (17.42)	4337 (0.79)
AGC^i^	99 (0.02)	18 (0.80)	29 (0.77)	13 (0.57)	30 (1.83)	189 (0.03)
Missing but positive for high-risk HPV	779 (0.14)	99 (4.42)	231 (6.13)	159 (6.98)	81 (4.93)	1349 (0.24)
ASC-H^j^	902 (0.17)	147 (6.56)	363 (9.63)	163 (7.16)	145 (8.83)	1720 (0.31)
AGC-FN^k^	7 (0.00)	4 (0.18)	9 (0.24)	3 (0.13)	6 (0.37)	29 (0.01)
HSIL^l^	411 (0.08)	185 (8.25)	443 (11.75)	233 (10.23)	215 (13.09)	1487 (0.27)
Carcinoma	1 (0.00)	1 (0.04)	10 (0.27)	2 (0.09)	3 (0.18)	17 (0.00)
**Histopathological diagnosis, n (%)**						
NILM	542,002 (100.00)	0 (0.00)	0 (0.00)	0 (0.00)	0 (0.00)	542,002 (98.20)
CIN1^m^	0 (0.00)	1542 (68.78)	2456 (65.15)	1445 (63.43)	948 (57.73)	6391 (1.16)
CIN2^n^	0 (0.00)	22 (0.98)	29 (0.77)	15 (0.66)	16 (0.97)	82 (0.01)
CIN2/3^o^	0 (0.00)	551 (24.58)	1113 (29.52)	716 (31.43)	485 (29.54)	2865 (0.52)
CIN3^p^	0 (0.00)	16 (0.71)	19 (0.50)	13 (0.57)	13 (0.79)	61 (0.01)
Carcinoma	0 (0.00)	111 (4.95)	153 (4.06)	89 (3.91)	180 (10.96)	533 (0.10)

a CCP: cervical cancer prevention.

b HPV: human papillomavirus.

c high-risk HPV: HPV-16/18/31/33/35/39/45/51/52/56/58/59/66/68.

d low-risk HPV: HPV-11/40/42/43/44/6/61/72/81.

e possible high-risk HPV: HPV-53/70/73/82/83.

f NILM: negative for intraepithelial lesion or malignancy.

g ASC-US: atypical squamous cells of undetermined significance.

h LSIL: low-grade squamous intraepithelial lesion.

i AGC: atypical glandular cells.

j ASC-H: atypical squamous cells, cannot exclude high-grade squamous intraepithelial lesion.

k AGC-FN: AGC-favor neoplastic.

l HSIL: high-grade squamous intraepithelial lesion.

m CIN1: cervical intraepithelial neoplasia grade 1.

n CIN2: cervical intraepithelial neoplasia grade 2.

o CIN2/3 is reflective of CIN2 or CIN3, i.e., HSIL; carcinoma consists of AIS (adenocarcinoma in situ) and cancer.

p CIN3: cervical intraepithelial neoplasia grade 3.

### Data-Driven Subgroups

**Figure 2.**
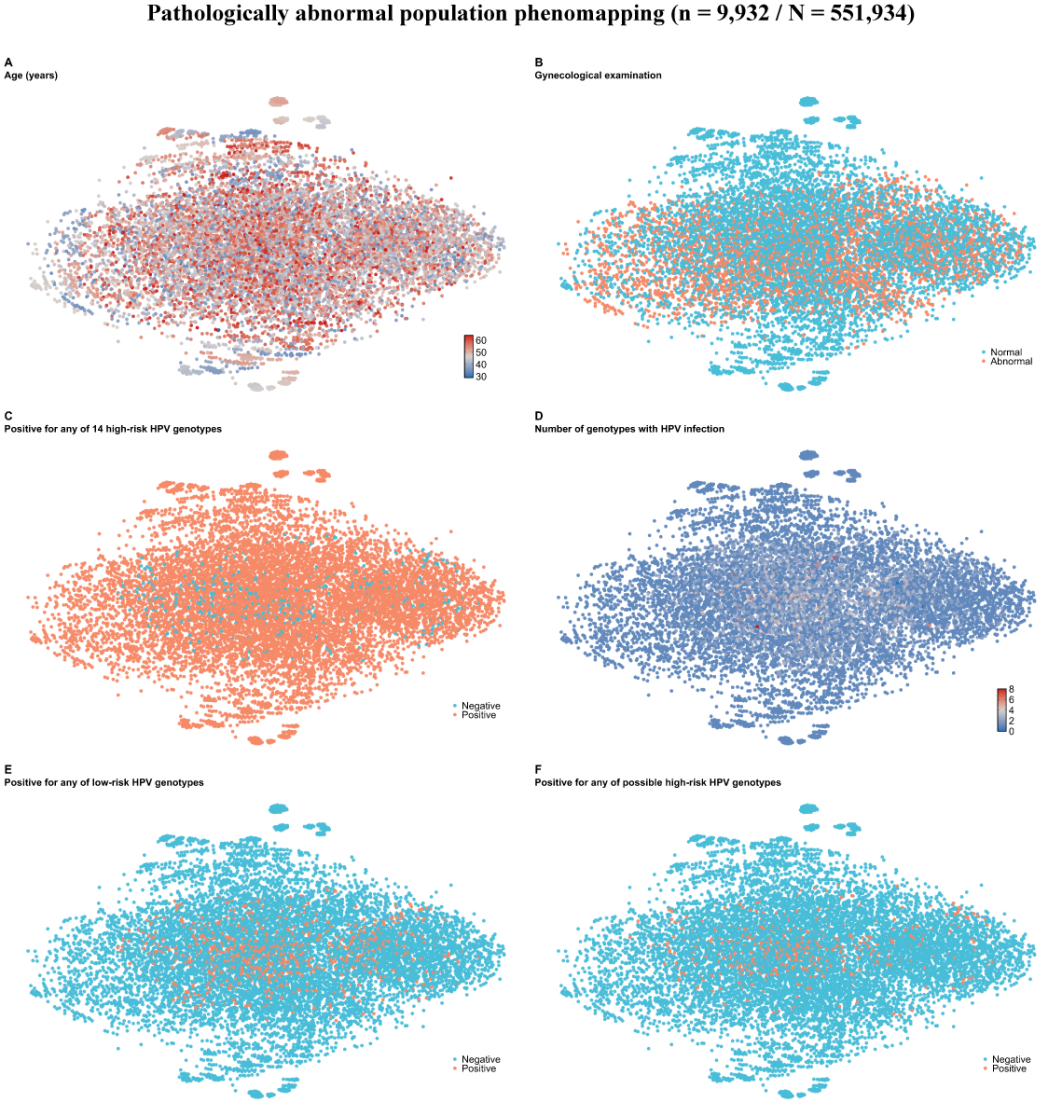
Manifold representations of the phenotypic architecture of 9932 individuals pathologically diagnosed with cervical abnormality from the discovery cohort (N=551,934). A total of 9932 individuals are embedded in the phenotypic space based on dissimilarity metrics (Gower’s distance) derived from 31 included phenotypic features; thus, phenotypically similar women tend to be topologically closer. In the subfigures, each dot represents an individual, with coloring based on the value of features. Since the dimensionality reduction is nonlinear, axes have been omitted, and only the comparisons between distances are meaningful. Pathologically abnormal diagnoses consisted of CIN1, CIN2, CIN3, and carcinoma. 14 high-risk HPV: HPV-16/18/31/33/35/39/45/51/52/56/58/59/66/68; low-risk HPV: HPV-11/40/42/43/44/6/61/72/81; possible high-risk HPV: HPV- 53/70/73/82/83.

**Figure 3.**
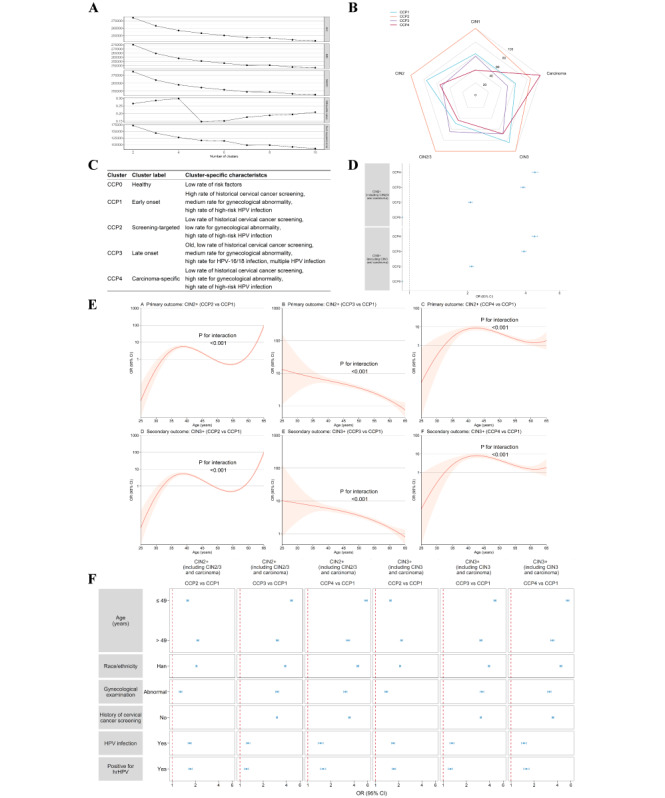
Determination of the 5 CCP subgroups with differential risks of CIN2+/CIN3+. (**A**) Elbow plot to determine the optimal number of clusters. Across 3 algorithms (k-means, LCA, and UMAP), identified CCP subgroups were stable, with the optimal number of clusters being 4 in 9932 women pathologically diagnosed with cervical abnormality. Women with normal screening test findings were considered as one independent CCP subgroup free of cervical cancer (Table 1). (**B**) Relative prevalence of pathologically abnormal diagnoses across CCP subgroups. For each pathologically abnormal diagnosis, the highest prevalence was designated as 100%, and the prevalence in each of the other CCP subgroups was relative to that prevalence (0‐100). (**C**) Characteristics of CCP subgroups. (**D**) CCP-specific risk estimates of CIN2+/CIN3+ compared with the CCP1 subgroup. Adjusted ORs (dots) and 95% CIs (error bars) are presented. The dashed line marks an OR of 1.00; lower limits of 95% CIs with values greater than 1.00 indicate significantly increased risk. (**E**) The CCP-specific risks of CIN2+/CIN3+ across age compared with the CCP1 subgroup. Age was transformed into a restricted cubic spline function for the analyses. *P* value was based on 2-sided Chi-squared test on the interaction between age and CCP subgroups. A *P* value of <.05 suggests that age modifies the association between CCP subgroups and CIN2+/CIN3+. Light-colored bands represent 95% CIs. (**F**) Subgroup analyses of CCP-specific risk estimates of CIN2+/CIN3+ compared with the CCP1 subgroup. Adjusted ORs (dots) and 95% CIs (error bars) are presented. The dashed line marks an OR of 1.00; lower limits of 95% CIs with values greater than 1.00 indicate significantly increased risk. Empty subfigures suggest insufficient samples for the analysis. AIC: Akaike information criterion; BIC: Bayesian information criterion; SABIC: sample size-adjusted BIC; CCP: cervical cancer prevention; CIN1: cervical intraepithelial neoplasia grade 1; CIN2: cervical intraepithelial neoplasia grade 2; CIN3: cervical intraepithelial neoplasia grade 3; CIN2+: cervical intraepithelial neoplasia grade 2 or worse; CIN3+: cervical intraepithelial neoplasia grade 3 or worse; OR: odds ratio; hrHPV: high-risk human papillomavirus.

The discovery cohort was extensively phenotyped based on 31 features (Figure 2). Visual assessment of the risk phenomaps revealed that nearly all 6 features were heterogeneously distributed in the phenomic space to varying degrees, indicating distinct phenotypic neighborhoods. In the discovery cohort, the CCP subgroups identified across 3 algorithms and all metrics were stable (Figure 3A and Table S4 in Multimedia Appendix 1), with the optimal number of clusters being 4 in the subpopulation of 9932 women with cervical abnormalities. The distribution of cervical abnormalities across subgroups is presented in Figure 3B. Additionally, women with normal screening results were treated as another CCP subgroup (Table 1). Following a detailed analysis of each subgroup’s features, we labeled the 5 identified subgroups as follows: (0) healthy, (1) early onset, (2) screening-targeted, (3) late onset, and (4) carcinoma-specific. CCP1 had the highest prevalence of CIN1, while CCP4 had the highest prevalence of carcinoma (Figure 3C). Most features were able to discriminate well between the subgroups. In the external cohort, the subgroups were consistent (Table S3 in Multimedia Appendix 1), though CCP1 was not identified due to missing information on previous screenings. The distribution of subgroups was similar across the discovery and external cohorts, with CCP0 being the most common subgroup and CCP4 having the highest prevalence of carcinoma.

### Risk Estimates and Diagnostic Validity

In the diagnostic validity analysis of the discovery cohort (Figure 3D), women in CCP2-4 exhibited a significantly increased risk of both CIN2+ (CCP2: OR 2.07, 95% CI [2.03‐2.12]; CCP3: 3.88 [3.78‐3.97]; CCP4: 4.47 [4.33‐4.63]) and CIN3+ (2.10 [2.05‐2.14]; 3.92 [3.82‐4.02]; 4.45 [4.31‐4.61]) compared to CCP1. Risk analysis across age groups showed that the risks for CIN2+/CIN3+ were evident in the age ranges of 34‐47 and 60‐65 in CCP2, 26‐61 in CCP3, and 35‐60 in CCP4, respectively (Figure 3E). Interaction analyses between age and subgroups revealed that the risks of CIN2+/CIN3+ increased with age in the 34‐39 and 60‐65 age ranges in CCP2, in the 35-43/44 age range in CCP4. Conversely, the risks decreased with age in CCP2 within the 40‐47 age range and in CCP4 within the 45‐60 age range (all *P* for interaction <.001). Subgroup analyses indicated that the risks for CIN2+/CIN3+ were present across nearly all subgroups, categorized by age, race, gynecological examination, screening history, hrHPV infection, and number of infections (Figure 3F and Table S5 in Multimedia Appendix 1).

**Figure 4.**
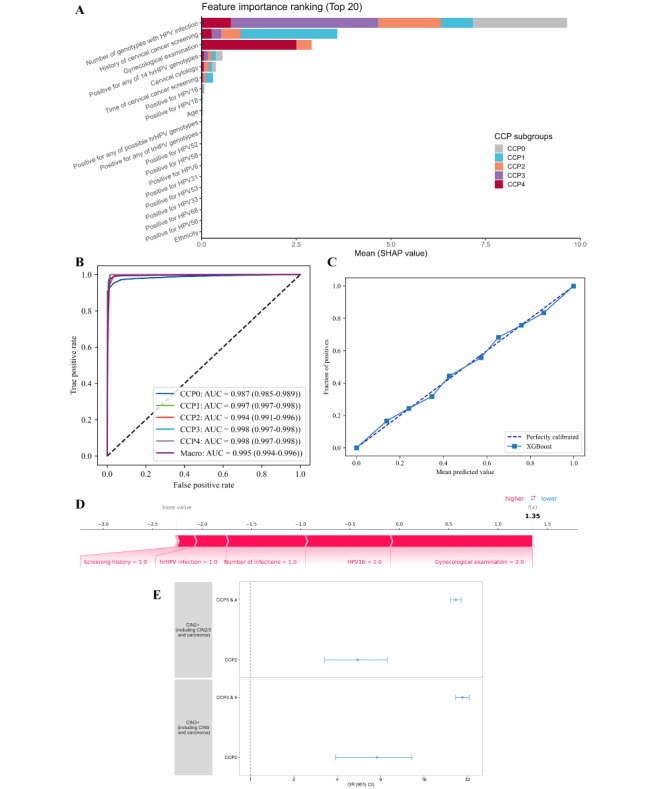
Feature importance, discrimination, calibration, explainability, and external diagnostic validity. (**A**) The top 20 features for prediction of CCP subgroups with differential risks of CIN2+/CIN3+ are shown. The y-axis represents the features included in the model development (in descending order of importance), and the x-axis indicates the mean of SHAP values. The 10 most important features were selected to train the final parsimonious model. (**B**) ROC curves in the internal validation from the discovery cohort. The dark purple line represents the macro-average of all 5 CCP subgroups. (**C**) Calibration curve of the alignment between predicted and observed CCP subgroups for the final model. The solid line corresponds to the calibration curve, with the dashed line corresponding to a reference for perfect calibration (ie, perfect alignment between the predicted and observed probabilities). (**D**) Explanation of the SCREENing tool (ie, the inference process of the final model with a woman in the CCP4 subgroup). (**E**) In external validation, to assess cross-cluster and cross-dataset risk differences, the model was measured and compared by diagnostic validity (CCP-specific ORs) between datasets. CCP: cervical cancer prevention; hrHPV: high-risk human papillomavirus; lrHPV: low-risk human papillomavirus; SHAP: SHapley Additive exPlanations; AUC: area under receiver operating characteristics curve; XGBoost: eXtreme gradient boosting.

SHAP analysis identified the top 10 key features for prediction, including the number of infections, screening history, gynecological examination, hrHPV infection, cervical cytology, time since previous screening, HPV-16/18 infection, age, and possible hrHPV infection (Figure 4A). The final XGBoost model demonstrated excellent discrimination (OVR AUROC 0.995 [0.994‐0.996]), with CCP0 showing the lowest AUROC of 0.987 [0.985‐0.989], and strong calibration (Brier score 0.021 [0.020‐0.022]) (Figure 4B–C and Table S6 in Multimedia Appendix 1). For the external diagnostic validity of the identified subgroups, women in CCP2-4 showed differential and increased risk for both CIN2+ (CCP2: 5.54 [3.27‐8.86]; CCP3&4: 26.56 [24.44‐28.88]) and CIN3+ (7.53 [3.90‐13.18]; 29.47 [26.46‐32.86]) compared to CCP0. The cross-cluster risk differences were consistent across the discovery and external cohorts. To illustrate the model’s explainability, Figure 4D presents the SHAP plot for a woman in the CCP4 subgroup, who had no previous screening and tested positive for HPV-16 and CIN1 based on cervical cytology. Given this information, our model predicted an elevated risk of CCP4. Additionally, a screenshot of our browser-accessible tool based on the model developed in this study, to clarify how the model can be easily accessed via a browser in clinical settings, is shown in Figure S1 in Multimedia Appendix 1.

### Developed Pathways

Based on our analyses, we proposed a triple SCREENing strategy to prioritize CCP subgroups with varying CIN2+/CIN3+ risks: (1) top priority for colposcopy referrals to women in CCP3-4 in resource-constrained settings, (2) higher priority given for scaling up organized, population-based HPV screening programs (with adequate follow-up) for women in CCP1-2, and (3) lowest priority for CCP0 women, with large-scale screening limited to resource-rich settings. To assess its impact, we evaluated the real-world usability and effectiveness of SCREENing. Sample clinicians reported that the identified subgroups and included features were clinically relevant for risk-based cervical cancer management, particularly for colposcopy referrals in CCP3-4. They also found the strategy transparent, interpretable, and generalizable across clinical settings, with the browser-accessible model feasible and effective for indicating risk probabilities during consultations. Sample epidemiologists highlighted the strategy’s potential to advance screening practices, public health policies, and trial design by enabling phenomapping, estimating subgroup-specific risk profiles, and establishing high-performance, cost-effective CCP for resource-limited regions. They noted its capability for testing effectiveness and cost-effectiveness in well-designed, prospective studies.

## Discussion

This study represents one of the largest EHR analyses to date, employing diverse machine learning methods and robust validation approaches to classify subgroups and predict CIN2+/CIN3+ risk. By leveraging comprehensive and interconnected phenotypic features, our study identified 5 CCP subgroups with varying risks. CCP2–4 had significantly higher risks of CIN2+ (CCP2: OR 2.07 [95% CI: 2.03‐2.12], CCP3: 3.88 [3.78‐3.97], CCP4: 4.47 [4.33‐4.63]) and CIN3+ (CCP2: 2.10 [2.05‐2.14], CCP3: 3.92 [3.82‐4.02], CCP4: 4.45 [4.31‐4.61]) compared to CCP1 (*P*<.001), consistent with the direction of results observed in the external cohort. Our findings offer a robust foundation for the proposed triple SCREENing strategy. This tailored approach prioritizes high-risk subgroups, providing actionable insights for cervical cancer prevention, particularly in LMICs.

To this end, various approaches, including single-cell transcriptomic analysis [49], sequence and phylogenetic analysis [50,51], and cluster analysis [48,52-54], have been employed to define genetic and cellular heterogeneity [55] in cervical cancer. These studies suggest that distinct subpopulations exhibit heterogeneous risk effects, linking individual features to varying absolute cervical cancer risk. Our 5 CCP subgroups align with findings from 2 major population-based clustering studies [56,57]. One study used a Poisson regression–based CEM clustering algorithm [56] to identify clusters of Indian states with similar cervical cancer incidence patterns. However, due to its focus on approximating missing data on sexual behavior, HPV prevalence, or cervical cancer incidence [56], the clusters were not ideal for screening purposes. That study did not provide sufficient details to estimate cluster-specific risks, and its reliance on features not typically available in routine screening limited real-world applicability. Additionally, strict model assumptions and the inclusion of relatively few features further constrained its generalizability. Another study performed hierarchical clustering of HPV-related methylation sites to identify subgroups of patients with cervical cancer [57]. While informative for prognosis and clinical management, the 3 clusters were not designed for cervical cancer screening. Instead, they are more suitable for guiding prognosis assessment, refining risk stratification, and optimizing treatment strategies in clinical practice.

In contrast, our study, specifically designed for cervical cancer screening, identified complex interactions among factors such as the number of infections, screening history, gynecological examination, hrHPV and HPV-16/18 infections, cervical cytology, time since previous screening, age, and possible hrHPV infection as key determinants of the 5 CCP subgroups with varying CIN2+/CIN3+ risks. Compared to previous studies [56,57], our design and identified subgroups are more representative of real-world cervical cancer screening. Leveraging the comprehensive and interconnected phenotypic features from EHRs, our algorithm extracted CCP-specific risk estimates, providing a robust foundation for the proposed triple SCREENing strategy. This strategy prioritizes subgroups with differential CIN2+/CIN3+ risks, offering a tailored approach to CCP. We recommend implementing SCREENing as a supplemental tool to existing guidelines [58-60], while accounting for the unique priorities and constraints of LMICs. SCREENing enables clinicians to perform effective, risk-based screening, followed by adequate diagnosis, surveillance, and management, while empowering policy makers to optimize public health policies and resource allocation. This approach has the potential to mitigate resource shortages in LMICs, reduce delays in diagnosis and treatment, and enhance screening efficiency by focusing efforts on high-risk populations, ultimately maximizing population-level benefits. Additionally, our analysis highlights the modifying effect of age on CCP-specific CIN2+/CIN3+ risks, emphasizing the need for greater attention to menopause and age [61] in cervical cancer screening strategies.

Methodologically, our study advances the external validation of machine learning algorithms for identifying subgroups and predicting cervical precancer or cancer risk using large-scale routine EHRs—a rarity in previous studies, which have often been limited to small samples. Our robust, structured framework of internal, external, and diagnostic validation enhances the acceptability and generalizability of unsupervised and supervised machine learning approaches in routine CCP and is adaptable to other diseases. The identified subgroups demonstrated good accuracy and diagnostic validity for CIN2+/CIN3+ both within and across datasets, though performance was lower in scenarios with missing key features. Differences in diagnostic validity across datasets may reflect variations in HPV infection patterns and previous screening histories, which influence the distribution of risk factors. Our findings of 5 subgroups and the proposed strategy are novel, offering a framework for assessing validity in screening, follow-up surveillance, and treatment for cervical cancer. This study signals the potential for more effective and targeted approaches to CCP.

This study represents one of the largest EHR analyses to date, employing multiple machine learning methods, datasets, and validation approaches to classify subgroups and predict CIN2+/CIN3+ risk. However, there are several limitations. First, a major limitation of this study is that our models were not externally validated beyond China. HPV infection patterns and the epidemiology of cervical abnormalities differ across countries and regions, which may impact the generalizability of our models. Given this variability, and although we used data from 5 distinct regions within China, we strongly recommend further validation of our models’ performance before applying them in settings not included in this study. We acknowledge that externally validating the model in additional multicenter studies worldwide is crucial for assessing its transferability and applicability across different clinical settings. Additionally, the tool may face challenges in fully adapting to various real-world scenarios when implemented outside the controlled validation environment of this study. To address these concerns, we plan to conduct independent external and prospective validations, as well as pilot implementation across diverse clinical settings in future studies. These efforts will evaluate the model’s effectiveness using a wider range of data, particularly in the context of real-world cervical cancer prevention across regions beyond those included in this study. We also encourage independent researchers to validate our model in their own settings, where feasible. While this study marks a significant step in the development of the SCREENing tool, we acknowledge that further research and validation are necessary to establish its effectiveness in real-world applications.

Second, we acknowledge that the use of retrospective EHR data may introduce biases and may not fully capture the real-time challenges of clinical practice. These challenges include issues such as missing data, data errors, and the heterogeneity of EHR systems across different settings. Although we made efforts to control for the quality of the EHR data, it remains inherently difficult to fully address these concerns without significant infrastructure changes, as well as ongoing monitoring and data validation. Therefore, we explicitly caution that the findings of this study should be interpreted with caution. In light of this limitation, future studies should aim to bridge the gap between retrospective analyses and the practical challenges of data collection in clinical settings. Third, while this study focused on routine EHR data, we acknowledge that incorporating multi-omics data—such as genomic, proteomic, and imaging data—could further enhance the model’s robustness and performance, providing valuable new insights into cervical abnormalities. We propose this as an area for future research to better understand the progression of cervical cancer and to offer novel perspectives on controlling and eliminating cervical cancer. Fourth, due to the limitations of routinely collected EHR content, we were unable to include several key sexual behavior features, such as the number of sexual partners and oral contraceptive use [62], in our analyses. These factors are known to significantly influence HPV infection and cervical cancer risk. While the model in this study demonstrated high performance, the absence of such data constrained our ability to further analyze the impact of sexual behaviors on findings, such as subgroup characterization, which could provide valuable insights. We recommend that future studies incorporate these data to validate our findings and offer a more comprehensive understanding of this field.

In conclusion, this study underscores the potential of leveraging machine learning algorithms and large-scale routine EHRs to enhance cervical cancer prevention strategies. By identifying key determinants of CIN2+/CIN3+ risk and classifying 5 distinct subgroups, our study provides a robust, data-driven foundation for the proposed triple SCREENing strategy. This approach prioritizes tailored prevention efforts for subgroups with varying risks, offering a novel and scalable tool to complement existing cervical cancer screening guidelines. Future work should focus on independent external and prospective validation to maximize the global impact of this strategy.

## Supplementary material

10.2196/67840 Multimedia Appendix 1 Data Supplement.

## Abbreviations

CCP: cervical cancer prevention
CIN: cervical intraepithelial neoplasia
EHR: electronic health record
HPV: human papillomavirus
hrHPV: high-risk HPV
LMIC: low- and middle-income country
OR: odds ratio
OVR AUROC: one-versus-rest area under the receiver operating characteristics curve
SCREENing: clinical Subgroups for CeRvical cancEr prEvention using computational pheNomaps and machine learnING
SHAP: SHapley Additive exPlanations
UMAP: uniform manifold approximation and projection
WHO: World Health Organization
XGBoost: eXtreme gradient boosting
