# Supplementary material for: Identifying Data-Driven Clinical Subgroups for Cervical Cancer Prevention With Machine Learning: Population-Based, External, and Diagnostic Validation Study
Source: JMIR Public Health Surveill. 2025 Mar 19;11:e67840. doi: 10.2196/67840 (PMC11939026; doi:10.2196/67840)
Supplement: Multimedia Appendix 1 [file publichealth-v11-e67840-s001.docx]

Table of Contents

[Appendix S1. Supplementary Methods 2](#_Toc187521960)

[Table S1. Glossary of terms of statistics and machine learning used in the study 5](#_Toc187521961)

[Table S2. Feature pre-processing 7](#_Toc187521962)

[Table S3. Characteristics by cervical cancer prevention (CCP) subgroups in external cohort 8](#_Toc187521963)

[Table S4. Detailed clustering process of LCA and metrics 11](#_Toc187521964)

[Table S5. Subgroup analyses of the CCP-specific risk estimates (ORs and 95% CIs) of the primary and secondary outcomes 12](#_Toc187521965)

[Table S6. Discrimination and calibration of SCREENing tool 13](#_Toc187521966)

[Figure S1. A guide to using the browser-accessible tool based on the model developed in this study 14](#_Toc187521967)

[Reference 15](#_Toc187521968)

# Appendix S1. Supplementary Methods

**Data source**

Deidentified data were retrieved from EHRs of national cervical cancer screening program in Fujian Province (discovery cohort, 2014-2022), China. In the national cervical cancer screening program (see http://www.nhc.gov.cn/fys/), each participant was interviewed using questionnaires, underwent a pelvic examination, and underwent genotyping for HPV infections. Colposcopy was performed for women who were tested positive for HPV-16/18 infection, while those positive for infections with the other high-risk HPV genotypes underwent further testing with ThinPrep cytology (Hologic), followed by colposcopy for those with abnormal cytological examination results. The inclusion criteria for participation in the national cervical cancer screening program were as follows: (1) aged 25 years or older; (2) having been sexually active for at least one year; and (3) without pregnancy. The exclusion criteria for participation in the program were as follows: (1) with a history of reproductive tract malignancy; (2) with a history of hysterectomy, cervical surgery, or pelvic radiation therapy. We included eligible women aged 25-65 years who took part in the cervical cancer screening. We used data from Fujian Province (2014-2022) to form a discovery cohort. The remaining EHR data from another five regions (comprising of Shenzhen City, Foshan City, Hubei Province, Gansu Province, and Guizhou Province) were used as the external validation cohort to assess the generalizability of the findings to distinct populations across China. This study was approved by the Ethics Committee of Fujian Maternal and Child Health Hospital (2023KY141) and obtained informed consent exemption.

**Study design**

We explored the data-driven CCP subgroups by applying unsupervised machine learning to the deeply phenotyped, population-based discovery cohort (Figure 1A-B). After identifying the CCP subgroups, we extracted CCP-specific risks for the outcomes of interest, conditioning on all predefined and algorithmically selected features, through weighted logistic regression analyses, which provided average odds ratio (OR) estimates (Figure 1C). Additionally, we stratified individuals based on key features for subgroup analyses. Finally, we trained a supervised machine learning model and developed pathways to classify individuals using the features most consistently linked to CCP subgroups, and evaluated its diagnostic validity and usability in the external cohort (Figure 1D).

**Features**

We specified a set of available features, which were selected on the basis of previous research findings [1-6] on cervical cancer and clinical (BD, HC, TY, XT, JW, SL, XH, ZZ, HX, SX, and PS), biostatistical (ZL, JW, and SL), and epidemiological (TT, BW, and HZ) expertise; features included demographic characteristics (age, race/ethnicity), cervical cancer screening history (year at current screening, history and time of cervical cancer screening), HPV infection, and medical examination results (gynecological examination and cytological examination results). The gynecological examination involved the visual inspection of the vulva, internal speculum examination of the vagina and cervix, and bimanual palpation of the adnexa and uterus. We also considered infection of several microorganisms in the vaginal microenvironment, including Trichomonas vaginitis, vulvovaginal candidiasis, and bacterial vaginosis. All features are listed in Table S2. Notably, 14 high-risk HPV genotypes [1-3] in this study consisted of HPV-16/18/31/33/35/39/45/51/52/56/58/59/66/68; low-risk HPV [1, 2] in this study consisted of HPV-11/40/42/43/44/6/61/72/81; possible high-risk HPV [1, 2] in this study consisted of HPV-53/70/73/82/83.

**Data pre-processing**

***Missing data***

We reviewed clinically relevant features, all with less than 2% missing data (see Table S2). Given the minimal missingness for key features, we created binary missing data indicators for each feature with missing values. This approach effectively captures both observed and missing data patterns [7]. Specifically, for each feature with missing values, we generated an indicator variable, where observations with missing value were assigned a value of 1, and observations with present values were assigned a value of 0. These newly created indicator variables reflect the missing data patterns across all features. This method aligns with that used in a paper [7] published in Lancet Rheumatology and is a common technique in machine learning contexts. These indicator variables, along with their corresponding features, were included in the subsequent analyses. We believe this approach helps assess and model inherent patterns in missing data during the training of our machine learning models. The performance of our model further supports the reliability and effectiveness of this approach.

***Feature pre-processing and selection***

Following missing data handling, we performed min-max scaling [8] to normalize continuous features in the 0 to 1 range, and encoded categorical features to numerical values using the one-hot encoding method [9]. To avoid collinearity of continuous features, we calculated pairwise correlations across features, and wherever pairs exceeded an absolute correlation coefficient of 0.7, we excluded the feature with the largest mean absolute correlation across all pairwise comparisons (Table S2). We also excluded categorical variables with near-zero variance (frequency cut-off for ratio of most common value to the second most common value of 99.9 to 0.1), resulting in 31 included features.

**Defining a computational phenomap**

***Dissimilarity distance calculation using Gower’s method***

We first computed a dissimilarity index that classified individuals based on their detailed characteristics according to Gower’s distance [10]. Gower’s method computes a distance value for each pair of individuals. For continuous features, Gower’s distance represents the absolute value of the difference between a pair of individuals divided by the range across all individuals. For categorical features, the method assigns “1” if the values are identical and “0” if they are not. Gower’s distance is ultimately calculated as the mean of these terms (Table S1).

***Visualization using uniform manifold approximation and projection (UMAP)***

To visualize phenotypic variation within the population and across neighbourhoods, we applied uniform manifold approximation and projection (UMAP) [11] for dimensionality reduction. UMAP is a non-linear method that generates a low-dimensional projection of processed data while preserving the fuzzy topological structure [11], both locally and globally. This technique allows for the interpretation of individual distributions within the multidimensional phenotypic space, based on the full range of baseline phenotypes. We set two key visualization parameters—the number of neighbors and the minimum distance—at 260 and 0.6, respectively. These values were selected to optimize the ability of UMAP to learn the manifold structure of the data and preserve the broader topological structure of our dataset while ensuring effective visualization [12]. It is important to note that these parameters affect only the visualization, not the underlying topological structure of the data.

**Clustering by unsupervised machine learning**

***k-means clustering***

For k-means clustering, we used the dissimilarity matrix based on Gower’s distance and the elbow method to determine the optimal number of CCP subgroups. The cluster centroids were initialized randomly, ensuring that they were maximally spaced from one another [13]. Clusters were then formed iteratively such that the Euclidean distance between any object and its assigned cluster centroid was at least as small as the distance between the object and any other centroid. In each iteration, objects were reassigned to the cluster with the closest centroid, and the centroids were recalculated. This process continued until no further improvements were observed, or the maximum number of 100 iterations was reached [14].

The elbow method was employed to select an appropriate number of clusters, as k-means clustering does not inherently provide this value [15]. The method computes the total within-cluster sum of squares error (SSE) for each candidate number of clusters. The SSE is plotted against the number of clusters, and the “elbow” point on the curve is used to determine the optimal number of clusters.

***LCA-derived clustering***

LCA is a finite mixture modeling techniques that enables researchers to determine whether unobserved subgroups exist within a population [16]. LCA models are based on the assumption that unobserved variables divide a population into mutually exclusive and collectively exhaustive latent classes [17]. We constructed multiple models for different class numbers, compared their fit, and selected the model that satisfied the following criteria: i) lower values of the BIC [18], the SABIC [19], and the Akaike information criteria (AIC) [20]; ii) a statistically significant result from the LoMendel-Rubin likelihood ratio test (LMR) [21], indicating that the model with k classes fits better than the model with k-1 classes; iii) average posterior probabilities of subgroup membership greater than or equal to 0.5 for each subgroup [22]; iv) the smallest class containing more than 5% of the total population [23]; and v) the highest silhouette value [24] (Figure 3A). For each individual, a posterior probability predicting the likelihood of belonging to each identified classes was estimated. A probability cutoff of greater than or equal to 0.5 was applied to assign each individual to a class, with the class having the highest posterior probability being assigned to that individual.

***UMAP-derived clustering***

To visualize phenotypic variation within the population and across neighbourhoods, we applied uniform manifold approximation and projection (UMAP) [11] for dimensionality reduction. UMAP is a non-linear method that generates a low-dimensional projection of processed data while preserving the fuzzy topological structure [11], both locally and globally. This technique allows for the interpretation of individual distributions within the multidimensional phenotypic space, based on the full range of baseline phenotypes. We set two key visualization parameters—the number of neighbors and the minimum distance—at 260 and 0.6, respectively. These values were selected to optimize the ability of UMAP to learn the manifold structure of the data and preserve the broader topological structure of our dataset while ensuring effective visualization [12]. It is important to note that these parameters affect only the visualization, not the underlying topological structure of the data.

***Optimal clustering achieved through consensus of three algorithms (k-means, LCA, and UMAP)***

We compared the optimal number of clusters identified using the three distinct methods: the elbow method for k-means clustering (Figure 3A), the five criteria for LCA-derived clustering (Figure 3A and Table S4), and the visualization of UMAP-derived clustering (Figure 2). Our goal was to determine the final optimal number of clusters that achieves the best performance across diverse metrics for all three algorithms, which is showed in Figure 3A. This process, referred to as the consensus of three algorithms in this study, ensures a robust evaluation. We believe that this rigorous approach enhances the reliability of the clustering results and enables precise identification of data-driven subgroups. Furthermore, the strong performance of our model underscores the effectiveness and reliability of this methodology.

**Defining** **phenotypically specific risk estimates**

We estimated the risk of each outcome by first building a subcohort of women. For each subcohort, multinomial logistic regression was built to estimate the probability of an individual belonging to the observed CCP subgroup (CCP0, CCP1, CCP2, CCP3, and CCP4) conditional on all predefined key features [25] denoted by $L$. The estimated probability ($P(group=observed CCP subgroup|L)$) was used as the propensity score to calculate the inverse probability weight (IPW) for average risk estimates within the cohort. The stabilized IPW was computed as $P(group=observed CCP subgroup)/P(group=observed CCP subgroup|L)$, where $L$ is the features, $P(group=observed CCP subgroup)$ is the group proportion within the cohort and served as the stabilization factor. To further reduce the influence of extreme weights, the stabilized weights were truncated at 30 [26, 27]. Only 31 individuals (31/551,934) with the stabilized weights greater than 30 were identified and then were truncated.

We then used weighted logistic models to estimate odds ratios (OR) of outcomes between the CCP subgroups after application of IPW. Additionally, we conducted analyses in subgroups based on age, race/ethnicity, history of cervical cancer screening, gynecological examination, and HPV infection. To further understand the association between CCP subgroups and outcomes across age, we conducted spline analyses, where age was treated as restricted cubic spline with knots placed at the 10th, 35th, 65th and 90th percentiles. We also performed interaction analyses between age and CCP subgroups to examine whether age modified the association between CCP subgroups and outcomes.

Robust sandwich variance estimators were used to provide an estimation of variance when applying weightings. In all analyses, evidence of statistical significance was considered when a 95% CI excluded unity. All analyses here were conducted using SAS Enterprise Guide v.7.1 (SAS Institute), and visualization of results was accomplished using R v.4.3.2.

**Developing and validating an eXtreme gradient boosting (XGBoost) algorithm to identify CCP subgroups**

***Data preprocessing***

For the training of the algorithm, categorical features with more than two levels were one-hot encoded and we removed features with near-zero variance (frequency cut-off for ratio of most common value to the second most common value of 99.9 to 0.1).

***Machine learning task creation***

We trained an eXtreme gradient boosting (XGBoost) algorithm [28] to predict five CCP subgroups with differential risks of outcomes of interest, using a subset of features, which was selected based on published studies, consensus of experts, and evaluation of feature importance, as per our previous work [29].

Specifically, the XGBoost algorithm was constructed to identify the phenotypes most strongly linked to the five personalized CCP subgroups. The model was trained in a randomly and proportionally selected subset of discovery cohort, consisting of 80% of the cohort, with model calibration done in 10% of the cohort and internal validation done in the remaining 10%. We calibrated the model using an isotonic regression algorithm. Optimal hyperparameters were tuned and selected using 10-fold cross-validation and Bayesian optimization [29, 30]. To assess feature importance and allow for interpretation of our model’s prediction, we used the SHapley Additive exPlanations (SHAP) method to identify a feature’s relative contribution, either positively or negatively, to the final prediction [29, 31]. Based on the ranking of feature importance, we selected a panel of key features, by comprehensive considerations on ready availability and accessibility in real-world clinical practice, to develop a simplified XGBoost model.

We used softmax function in the output layer of our machine learning model. Model performance was evaluated with multiclass cross-entropy loss [32] as the loss function, using AUROC (area under receiver operating characteristics curve), accuracy, F1 score, AUPRC (area under precision-recall curve) as evaluation metrics. We extended the AUROC as OVR (one-versus-rest) AUROC for multiple classes [33], which indicates model’s discrimination in general, by calculating an AUROC individually for each CCP subgroup class and then a macro-average ROC curve defined as the mean of true positive rates of each class as a function of false positive rate. Calibration performance was assessed by Brier score and calibration curve. We calculated 95% Cis by bootstrapping with 500 resamples. Additionally, to fully aid in the global understanding of the model and promote translation into real-world practical application, we also employed SHAP method to improve its explainability. Finally, diagnostic validation was performed by measuring CCP-specific ORs in external cohort and assessing cross-cluster and cross-dataset risk differences. The final model was named SCREENing (for clinical Subgroups for CeRvical cancEr prEvention using computational phenomaps and machine learnING) and an online browser-accessible version of SCREENing was also made available for external use. We adhered to the Transparent Reporting of a multivariable prediction model for Individual Prognosis or Diagnosis (TRIPOD) statement [34].

All analyses here were performed using Python v.3.11.6.

**Statistical analysis**

Categorical features were summarized as numbers (percentages), and continuous features as median (Q1, Q3), as appropriate. Continuous features among groups were compared using Kruskal-Wallis test, and categorical features were compared using chi-squared test. Pearson’s r was used to assess the pairwise correlation between continuous features. When extracting phenotypically specific risk estimates during the training stage, we applied weighted logistic models with robust sandwich variance estimators. To further understand the association between CCP subgroups and outcomes across age, we conducted spline analyses, where age was treated as restricted cubic spline with knots placed at the 10th, 35th, 65th and 90th percentiles. We also performed interaction analyses between age and CCP subgroups to examine whether age modified the association between CCP subgroups and outcomes. Additionally, we stratified women based on key features to conduct subgroup analyses. Findings were considered to be statistically significant when the 95% CIs for ORs on a relative scale excluded unity. A glossary of statistical and machine learning terms is included in Table S1. All analyses were conducted in Python (v.3.11.6), and SAS Enterprise Guide (v.7.1, SAS Institute Inc, Cary, NC) and R (v.4.3.2, R Foundation for Statistical Computing, Vienna, Austria).

# Table S1. Glossary of terms of statistics and machine learning used in the study

| Dissimilarity matrix | A numeric matrix that expresses the distance between pairs of individuals, based on a dissimilarity metric (we used Gower’s metric in this study). |
| --- | --- |
| Gower’s distance | A metric that measures the dissimilarity of two items based on mixed numeric and non-numeric data [10]. For continuous features, Gower’s distance represents the absolute value of the difference between a pair of individuals divided by the range across all individuals. For categorical features, the method assigns “1” if the values are identical and “0” if they are not. Gower’s distance is ultimately calculated as the mean of these terms. Gower’s distance is ultimately calculated as the mean of these terms for all available features. By calculating the average dissimilarity between all phenotypic features, Gower’s distance provides a metric that quantifies the similarity between all possible pairs of individuals in a population (ranging from 0 [most similar] to 1 [most dissimilar]). This provides an objective and unbiased away to identify groups of phenotypically similar patients in a population. |
| Uniform Manifold Approximation and Projection (UMAP) | An algorithm [11] for dimensionality reduction based on manifold learning techniques, simplicial complexes, and ideas from topological data analysis. |
| eXtreme gradient boosting (XGBoost) | A gradient boosting machine learning algorithm, that is based on decision trees and models. It constructs a graph that examines the input under various sequential "if" statements. The algorithm progressively adds more “if” conditions to the decision tree to improve the predictions of the overall model [28]. |
| Softmax function | A mathematical function that converts a vector of numbers into a vector of probabilities, where the probabilities of each value are proportional to the relative scale of each value in the vector. |
| Area under the receiver operating characteristic curve (AUROC) | AUROC is a popular evaluation metric for binary classification models. It measures the model's ability to discriminate between positive and negative samples across all possible classification thresholds. The AUROC ranges between 0 and 1, where a higher value indicates better discrimination performance.  The receiver operating characteristic (ROC) Curve is a graphical representation of a binary classification model's performance. It illustrates the trade-off between the true positive rate (sensitivity, TPR) and the false positive rate (1 – specificity, FPR) at various classification thresholds. The curve is created by plotting the true positive rate on the y-axis against the false positive rate on the x-axis.  TPR measures the proportion of actual positive samples that are correctly classified as positive by the model. It is calculated as TPR = TP / (TP + FN), where TP denotes true positives and FN denotes false negatives. FPR measures the proportion of actual negative samples that are incorrectly classified as positive by the model. It is calculated as FPR = FP / (FP + TN), where FP denotes false positives and TN denotes true negatives.  The AUROC represents the area under the ROC curve. It quantifies the overall performance of the model across all possible classification thresholds. The higher the AUROC value, the better the model's discriminatory ability. The calculation of AUROC involves integrating the ROC curve or approximating it using numerical methods. |
| OVR (one-versus-rest) AUROC | We extended the AUROC as OVR (one-versus-rest) AUROC for multiple classes [33], which indicates model’s discrimination in general, by calculating an AUROC individually for each CCP subgroup class and then a macro-average ROC curve defined as the mean of true positive rates of each class as a function of false positive rate. |
| Accuracy | Accuracy is a commonly used evaluation metric for classification models. It measures the proportion of correctly classified samples out of the total number of samples in the dataset. Accuracy provides a general measure of the model's correctness in predicting the class labels.  True Positives (TP): The number of samples that are correctly classified as positive by the model. True Negatives (TN): The number of samples that are correctly classified as negative by the model. False Positives (FP): The number of samples that are incorrectly classified as positive by the model. False Negatives (FN): The number of samples that are incorrectly classified as negative by the model. Total Number of Samples (N): The total number of samples in the dataset.  The accuracy (ACC) is calculated using the following formula: ACC = (TP + TN) / N. The numerator (TP + TN) represents the number of correctly classified samples, while the denominator (N) represents the total number of samples in the dataset. Dividing the numerator by the denominator yields the proportion of correctly classified samples, which is the accuracy. |
| F1 score | F1 score is a widely used evaluation metric for classification models, particularly in cases where there is an imbalance between the classes or when both precision and recall are equally important. The F1 score combines precision and recall into a single value, providing a balanced measure of a model's performance.  The F1 score is calculated using the following formula: F1 Score = 2 * (Precision * Recall) / (Precision + Recall). The formula combines precision and recall by taking their harmonic mean. The harmonic mean places more emphasis on lower values, ensuring that the F1 score is only high when both precision and recall are high.  The F1 score ranges between 0 and 1, with a higher value indicating better performance. |
| AUPRC (area under precision-recall curve) | A performance metric used to evaluate binary classification models, especially in cases where the classes are imbalanced. It focuses on the performance of the model with respect to precision and recall rather than accuracy, which can be misleading in the presence of class imbalance. |
| Brier score | Brier score is a widely used evaluation metric for probabilistic predictions in binary classification problems. It measures the mean squared difference between the predicted probabilities and the actual binary outcomes. The Brier score provides a measure of the calibration and accuracy of the predicted probabilities.  The formula to calculate the Brier score is as follows: $Brier Score=\left( \frac{1}{N} \right)*\sum\left( \left( p_{i}-o_{i} \right)^{2} \right)$. Here, N is the total number of samples, $p_{i}$ is the predicted probability for sample i, and $o_{i}$ is the corresponding binary outcome (0 or 1) for sample i.  The Brier score ranges between 0 and 1, with 0 indicating a perfect calibration and accuracy, and 1 indicating poor calibration and accuracy. A lower Brier score implies better predictive performance, indicating that the predicted probabilities are more calibrated and closer to the actual outcomes. |
| SHapley Additive exPlanations (SHAP) method | A method used to explain the output of machine learning models [35]. It is based on cooperative game theory, specifically the Shapley values, which provide a way to fairly distribute the payout among players depending on their contribution to the total payout. |

# Table S2. Feature pre-processing

| Feature name | Reason for exclusion |
| --- | --- |
| Age | Included |
| Race/ethinicity | Included |
| History of cervical cancer screening | Included |
| Time of cervical cancer screening | Included |
| Gynecological examination | Included |
| Positive for any of 14 high-risk HPV (hrHPV) genotypes | Included |
| Positive for any of low-risk HPV (lrHPV) genotypes | Included |
| Positive for any of possible hrHPV genotypes | Included |
| HPV16 | Included |
| HPV18 | Included |
| HPV31 | Included |
| HPV33 | Included |
| HPV35 | Included |
| HPV39 | Included |
| HPV45 | Included |
| HPV51 | Included |
| HPV52 | Included |
| HPV56 | Included |
| HPV58 | Included |
| HPV59 | Included |
| HPV66 | Included |
| HPV68 | Included |
| HPV11 | Included |
| HPV40 | Near-zero variance |
| HPV42 | Included |
| HPV43 | Included |
| HPV44 | Included |
| HPV6 | Included |
| HPV61 | Near-zero variance |
| HPV72 | Near-zero variance |
| HPV81 | Included |
| HPV53 | Included |
| HPV70 | Near-zero variance |
| HPV73 | Near-zero variance |
| HPV82 | Near-zero variance |
| HPV83 | Near-zero variance |
| Number of genotypes with HPV infection | Included |
| Cytological examination results | Included |

“Included” means these features will be included in the training of the extreme gradient boosting model based on availability.

# Table S3. Characteristics by cervical cancer prevention (CCP) subgroups in external cohort

| **Characteristics** | **CCP0**  **(N=42,245)** | **CCP2**  **(N=1,689)** | **CCP3**  **(N=1,644)** | **CCP4**  **(N=1,552)** | ***P* value** | **Total**  **(N=47,130)** |
| --- | --- | --- | --- | --- | --- | --- |
| Region |  |  |  |  | <.001 |  |
| Foshan | 1300 (3.08) | 292 (21.57) | 152 (9.21) | 206 (10.60) |  | 1950 (4.14) |
| Gansu | 1947 (4.62) | 325 (24.00) | 791 (47.94) | 1248 (64.23) |  | 4311 (9.15) |
| Guizhou | 390 (0.92) | 121 (8.94) | 142 (8.61) | 126 (6.48) |  | 779 (1.65) |
| Hubei | 871 (2.06) | 163 (12.04) | 238 (14.42) | 318 (16.37) |  | 1590 (3.37) |
| Shenzhen | 37675 (89.31) | 453 (33.46) | 327 (19.82) | 45 (2.32) |  | 38500 (81.69) |
| Age (years) | 37.00 (32.00, 45.00) | 40.00 (34.00, 48.00) | 39.00 (32.00, 50.00) | 40.00 (34.00, 49.00) | <.001 | 37.00 (32.00, 45.00) |
| Race/ethnicity |  |  |  |  | NA |  |
| Missing | 42245 (100.00) | 1689 (100.00) | 1644 (100.00) | 1552 (100.00) |  | 47130 (100.00) |
| History of cervical cancer screening |  |  |  |  | NA |  |
| Missing | 42245 (100.00) | 1689 (100.00) | 1644 (100.00) | 1552 (100.00) |  | 47130 (100.00) |
| Time of previous screening |  |  |  |  | NA |  |
| Missing | 42245 (100.00) | 1689 (100.00) | 1644 (100.00) | 1552 (100.00) |  | 47130 (100.00) |
| Gynecological examination |  |  |  |  | <.001 |  |
| Missing | 604 (1.43) | 335 (19.83) | 196 (11.92) | 0 (0.00) |  | 1135 (2.41) |
| Normal | 28216 (66.79) | 1354 (80.17) | 755 (45.92) | 0 (0.00) |  | 30325 (64.34) |
| Abnormal | 13425 (31.78) | 0 (0.00) | 693 (42.15) | 1552 (100.00) |  | 15670 (33.25) |
| Positive for high-risk HPV |  |  |  |  | <.001 |  |
| No | 39299 (93.03) | 25 (1.48) | 4 (0.24) | 0 (0.00) |  | 39328 (83.45) |
| Yes | 2946 (6.97) | 1664 (98.52) | 1640 (99.76) | 1552 (100.00) |  | 7802 (16.55) |
| Positive for low-risk HPV |  |  |  |  | <.001 |  |
| No | 41340 (97.86) | 1684 (99.70) | 1045 (63.56) | 1552 (100.00) |  | 45621 (96.80) |
| Yes | 905 (2.14) | 5 (0.30) | 599 (36.44) | 0 (0.00) |  | 1509 (3.20) |
| Positive for possible high-risk HPV |  |  |  |  | <.001 |  |
| No | 41544 (98.34) | 1669 (98.82) | 1318 (80.17) | 1552 (100.00) |  | 46083 (97.78) |
| Yes | 701 (1.66) | 20 (1.18) | 326 (19.83) | 0 (0.00) |  | 1047 (2.22) |
| Number of HPV infections | 0.00 (0.00, 0.00) | 1.00 (1.00, 1.00) | 2.00 (2.00, 3.00) | 1.00 (1.00, 1.00) | <.001 | 0.00 (0.00, 0.00) |
| Positive for HPV-16 |  |  |  |  | <.001 |  |
| No | 1998 (99.42) | 1167 (69.09) | 1133 (68.92) | 987 (63.60) |  | 45285 (96.09) |
| Yes | 247 (0.58) | 522 (30.91) | 511 (31.08) | 565 (36.40) |  | 1845 (3.91) |
| Positive for HPV-18 |  |  |  |  | <.001 |  |
| No | 41993 (99.40) | 1567 (92.78) | 1425 (86.68) | 1484 (95.62) |  | 46469 (98.60) |
| Yes | 252 (0.60) | 122 (7.22) | 219 (13.32) | 68 (4.38) |  | 661 (1.40) |
| Positive for HPV-31 |  |  |  |  | <.001 |  |
| No | 42107 (99.67) | 1631 (96.57) | 1526 (92.82) | 1489 (95.94) |  | 46753 (99.20) |
| Yes | 138 (0.33) | 58 (3.43) | 118 (7.18) | 63 (4.06) |  | 377 (0.80) |
| Positive for HPV-33 |  |  |  |  | <.001 |  |
| No | 42117 (99.70) | 1638 (96.98) | 1540 (93.67) | 1500 (96.65) |  | 46795 (99.29) |
| Yes | 128 (0.30) | 51 (3.02) | 104 (6.33) | 52 (3.35) |  | 335 (0.71) |
| Positive for HPV-35 |  |  |  |  | <.001 |  |
| No | 42192 (99.87) | 1677 (99.29) | 1602 (97.45) | 1540 (99.23) |  | 47011 (99.75) |
| Yes | 53 (0.13) | 12 (0.71) | 42 (2.55) | 12 (0.77) |  | 119 (0.25) |
| Positive for HPV-39 |  |  |  |  | <.001 |  |
| No | 42027 (99.48) | 1623 (96.09) | 1468 (89.29) | 1485 (95.68) |  | 46603 (98.88) |
| Yes | 218 (0.52) | 66 (3.91) | 176 (10.71) | 67 (4.32) |  | 527 (1.12) |
| Positive for HPV-45 |  |  |  |  | <.001 |  |
| No | 42185 (99.86) | 1676 (99.23) | 1585 (96.41) | 1536 (98.97) |  | 46982 (99.69) |
| Yes | 60 (0.14) | 13 (0.77) | 59 (3.59) | 16 (1.03) |  | 148 (0.31) |
| Positive for HPV-51 |  |  |  |  | <.001 |  |
| No | 41973 (99.36) | 1601 (94.79) | 1448 (88.08) | 1495 (96.33) |  | 46517 (98.70) |
| Yes | 272 (0.64) | 88 (5.21) | 196 (11.92) | 57 (3.67) |  | 613 (1.30) |
| Positive for HPV-52 |  |  |  |  | <.001 |  |
| No | 41295 (97.75) | 1397 (82.71) | 1117 (67.94) | 1274 (82.09) |  | 45083 (95.66) |
| Yes | 950 (2.25) | 292 (17.29) | 527 (32.06) | 278 (17.91) |  | 2047 (4.34) |
| Positive for HPV-56 |  |  |  |  | <.001 |  |
| No | 42049 (99.54) | 1610 (95.32) | 1461 (88.87) | 1502 (96.78) |  | 46622 (98.92) |
| Yes | 196 (0.46) | 79 (4.68) | 183 (11.13) | 50 (3.22) |  | 508 (1.08) |
| Positive for HPV-58 |  |  |  |  | <.001 |  |
| No | 41787 (98.92) | 1475 (87.33) | 1249 (75.97) | 1348 (86.86) |  | 45859 (97.30) |
| Yes | 458 (1.08) | 214 (12.67) | 395 (24.03) | 204 (13.14) |  | 1271 (2.70) |
| Positive for HPV-59 |  |  |  |  | <.001 |  |
| No | 42060 (99.56) | 1632 (96.63) | 1498 (91.12) | 1498 (96.52) |  | 46688 (99.06) |
| Yes | 185 (0.44) | 57 (3.37) | 146 (8.88) | 54 (3.48) |  | 442 (0.94) |
| Positive for HPV-66 |  |  |  |  | <.001 |  |
| No | 42126 (99.72) | 1640 (97.10) | 1523 (92.64) | 1526 (98.32) |  | 46815 (99.33) |
| Yes | 119 (0.28) | 49 (2.90) | 121 (7.36) | 26 (1.68) |  | 315 (0.67) |
| Positive for HPV-68 |  |  |  |  | <.001 |  |
| No | 42026 (99.48) | 1648 (97.57) | 1483 (90.21) | 1512 (97.42) |  | 46669 (99.02) |
| Yes | 219 (0.52) | 41 (2.43) | 161 (9.79) | 40 (2.58) |  | 461 (0.98) |
| Positive for HPV-11 |  |  |  |  | <.001 |  |
| No | 42190 (99.87) | 1687 (99.88) | 1604 (97.57) | 1552 (100.00) |  | 47033 (99.79) |
| Yes | 55 (0.13) | 2 (0.12) | 40 (2.43) | 0 (0.00) |  | 97 (0.21) |
| Positive for HPV-42 |  |  |  |  | <.001 |  |
| No | 41974 (99.36) | 1688 (99.94) | 1453 (88.38) | 1552 (100.00) |  | 46667 (99.02) |
| Yes | 271 (0.64) | 1 (0.06) | 191 (11.62) | 0 (0.00) |  | 463 (0.98) |
| Positive for HPV-43 |  |  |  |  | <.001 |  |
| No | 42120 (99.70) | 1689 (100.00) | 1553 (94.46) | 1552 (100.00) |  | 46914 (99.54) |
| Yes | 125 (0.30) | 0 (0.00) | 91 (5.54) | 0 (0.00) |  | 216 (0.46) |
| Positive for HPV-44 |  |  |  |  | <.001 |  |
| No | 42108 (99.68) | 1688 (99.94) | 1568 (95.38) | 1552 (100.00) |  | 46916 (99.55) |
| Yes | 137 (0.32) | 1 (0.06) | 76 (4.62) | 0 (0.00) |  | 214 (0.45) |
| Positive for HPV-6 |  |  |  |  | <.001 |  |
| No | 42136 (99.74) | 1688 (99.94) | 1553 (94.46) | 1552 (100.00) |  | 46929 (99.57) |
| Yes | 109 (0.26) | 1 (0.06) | 91 (5.54) | 0 (0.00) |  | 201 (0.43) |
| Positive for HPV-81 |  |  |  |  | <.001 |  |
| No | 41976 (99.36) | 1689 (100.00) | 1458 (88.69) | 1552 (100.00) |  | 46675 (99.03) |
| Yes | 269 (0.64) | 0 (0.00) | 186 (11.31) | 0 (0.00) |  | 455 (0.97) |
| Positive for HPV-53 |  |  |  |  | <.001 |  |
| No | 41644 (98.58) | 1670 (98.88) | 1369 (83.27) | 1552 (100.00) |  | 46235 (98.10) |
| Yes | 601 (1.42) | 19 (1.12) | 275 (16.73) | 0 (0.00) |  | 895 (1.90) |
| Cervical cytology examination |  |  |  |  | <.001 |  |
| NILM | 2446 (5.79) | 60 (3.55) | 152 (9.25) | 99 (6.38) |  | 2757 (5.85) |
| No examination due to negative for high-risk HPV | 38182 (90.38) | 0 (0.00) | 0 (0.00) | 0 (0.00) |  | 38182 (81.01) |
| ASC-US | 1138 (2.69) | 691 (40.91) | 647 (39.36) | 723 (46.59) |  | 3199 (6.79) |
| LSIL | 235 (0.56) | 550 (32.56) | 672 (40.88) | 384 (24.74) |  | 1841 (3.91) |
| AGC | 0 (0.00) | 12 (0.71) | 4 (0.24) | 10 (0.64) |  | 26 (0.06) |
| Missing but positive for high-risk HPV | 196 (0.46) | 192 (11.37) | 46 (2.80) | 112 (7.22) |  | 546 (1.16) |
| ASC-H | 46 (0.11) | 101 (5.98) | 67 (4.08) | 124 (7.99) |  | 338 (0.72) |
| AGC-FN | 0 (0.00) | 1 (0.06) | 0 (0.00) | 1 (0.06) |  | 2 (0.00) |
| HSIL | 2 (0.00) | 78 (4.62) | 56 (3.41) | 99 (6.38) |  | 235 (0.50) |
| Carcinoma | 0 (0.00) | 4 (0.24) | 0 (0.00) | 0 (0.00) |  | 4 (0.01) |
| Histopathological diagnosis |  |  |  |  | <.001 |  |
| NILM | 40071 (94.85) | 672 (39.79) | 630 (38.32) | 506 (32.60) |  | 41879 (88.86) |
| CIN1 | 1884 (4.46) | 770 (45.59) | 755 (45.92) | 586 (37.76) |  | 3995 (8.48) |
| CIN2 | 172 (0.41) | 125 (7.40) | 127 (7.73) | 137 (8.83) |  | 561 (1.19) |
| CIN2/3 | 26 (0.06) | 13 (0.77) | 44 (2.68) | 135 (8.70) |  | 218 (0.46) |
| CIN3 | 75 (0.18) | 89 (5.27) | 77 (4.68) | 146 (9.41) |  | 387 (0.82) |
| Carcinoma | 17 (0.04) | 20 (1.18) | 11 (0.67) | 42 (2.71) |  | 90 (0.19) |

Categorical features were summarized as numbers (percentages), and continuous features as median (Q1, Q3), as appropriate. Continuous features among groups were compared using Kruskal-Wallis test, and categorical features were compared using chi-squared test.

Abbreviations: CCP=cervical cancer prevention; HPV=human papillomavirus; high-risk HPV=HPV-16/18/31/33/35/39/45/51/52/56/58/59/66/68; low-risk HPV=HPV-11/40/42/43/44/6/61/72/81; possible high-risk HPV=HPV-53/70/73/82/83; NILM=negative for intraepithelial lesion or malignancy; ASC-US=atypical squamous cells of undetermined significance; CIN1= cervical intraepithelial neoplasia grade 1; LSIL=low-grade squamous intraepithelial lesion; AGC=atypical glandular cells; ASC-H=atypical squamous cells, cannot exclude high-grade squamous intraepithelial lesion; AGC-FN=AGC-favor neoplastic; HSIL=high-grade squamous intraepithelial lesion; CIN2= cervical intraepithelial neoplasia grade 2; CIN3= cervical intraepithelial neoplasia grade 3; CIN2/3 is reflective of CIN2 or CIN3, i.e., HSIL; carcinoma consists of AIS (adenocarcinoma in situ) and cancer.

# Table S4. Detailed clustering process of LCA and metrics

| Number of clusters | Sample size | Number of parameters | AIC | BIC | SABIC | Average posterior probabilities of subgroup membership | *P* value of LoMendel-Rubin likelihood ratio test (LMR) [21] |
| --- | --- | --- | --- | --- | --- | --- | --- |
| 2 | 9932 | 123 | 274132.46 | 275018.49 | 274627.62 | 22.59 | - |
| 3 | 9932 | 185 | 263224.05 | 264556.70 | 263968.79 | 22.59 | <.001 |
| 4 | 9932 | 247 | 256958.57 | 258737.84 | 257952.91 | 16.53 | <.001 |
| 5 | 9932 | 309 | 253236.98 | 255462.86 | 254480.91 | 2.66 | <.001 |
| 6 | 9932 | 371 | 250239.13 | 252911.63 | 251732.65 | 2.92 | <.001 |
| 7 | 9932 | 433 | 247301.56 | 250420.68 | 249044.67 | 3.48 | <.001 |
| 8 | 9932 | 495 | 246924.75 | 250490.49 | 248917.45 | 3.85 | <.001 |
| 9 | 9932 | 557 | 244304.96 | 248317.32 | 246547.26 | 3.89 | <.001 |
| 10 | 9932 | 619 | 243060.43 | 247519.40 | 245552.31 | 2.84 | <.001 |

# Table S5. Subgroup analyses of the CCP-specific risk estimates (ORs and 95% CIs) of the primary and secondary outcomes

| Characteristics | CIN2+ | | | CIN3+ | | |
| --- | --- | --- | --- | --- | --- | --- |
|  | CCP2 vs CCP1 | CCP3 vs CCP1 | CCP4 vs CCP1 | CCP2 vs CCP1 | CCP3 vs CCP1 | CCP4 vs CCP1 |
| Age (years) |  |  |  |  |  |  |
| ≤ 49 | 1.58 (1.54, 1.63) | 4.67 (4.51, 4.83) | 5.71 (5.46, 5.97) | 1.57 (1.52, 1.61) | 4.71 (4.55, 4.87) | 5.44 (5.20, 5.69) |
| > 49 | 2.14 (2.07, 2.22) | 3.05 (2.94, 3.17) | 3.33 (3.16, 3.51) | 2.18 (2.11, 2.25) | 3.09 (2.97, 3.21) | 3.45 (3.27, 3.63) |
| Race/ethnicity |  |  |  |  |  |  |
| Han | 2.06 (2.02, 2.11) | 3.88 (3.78, 3.98) | 4.47 (4.32, 4.62) | 2.09 (2.04, 2.13) | 3.93 (3.83, 4.03) | 4.45 (4.30, 4.60) |
| Gynecological examination |  |  |  |  |  |  |
| Abnormal | 1.28 (1.23, 1.34) | 3.04 (2.88, 3.21) | 3.08 (2.92, 3.25) | 1.38 (1.32, 1.44) | 3.18 (3.01, 3.36) | 3.15 (2.98, 3.33) |
| History of cervical cancer screening |  |  |  |  |  |  |
| No | NA (NA, NA) | 3.02 (2.97, 3.08) | 3.49 (3.39, 3.59) | NA (NA, NA) | 3.08 (3.02, 3.14) | 3.50 (3.40, 3.60) |
| HPV infection |  |  |  |  |  |  |
| Yes | 1.69 (1.60, 1.77) | 1.27 (1.20, 1.35) | 1.47 (1.36, 1.59) | 1.70 (1.62, 1.79) | 1.30 (1.22, 1.38) | 1.47 (1.37, 1.59) |
| Positive for high-risk HPV |  |  |  |  |  |  |
| Yes | 1.72 (1.63, 1.81) | 1.21 (1.14, 1.29) | 1.58 (1.46, 1.71) | 1.73 (1.64, 1.83) | 1.24 (1.16, 1.32) | 1.58 (1.46, 1.72) |

Subgroup analyses of CCP-specific risk estimates of CIN2+/CIN3+ compared with the CCP1 subgroup. Adjusted ORs and 95% CIs are presented. Lower limits of 95% CIs with values greater than 1.00 indicate significantly increased risk.

Abbreviations: CCP=cervical cancer prevention; CIN2+= cervical intraepithelial neoplasia grade 2 or worse; CIN3+= cervical intraepithelial neoplasia grade 3 or worse; OR=odds ratio; CI=confidence interval; HPV=human papillomavirus; NA=not available due to that insufficient samples made parameter estimation unavailable.

# Table S6. Discrimination and calibration of SCREENing tool

| Model | OVR AUROC | Accuracy | F1 score | AUPRC | Brier score |
| --- | --- | --- | --- | --- | --- |
| XGBoost | 0.995 (0.994-0.996) | 0.984 (0.983-0.985) | 0.545 (0.518-0.570) | 0.611 (0.588-0.640) | 0.021 (0.020-0.022) |

# Figure S1. A guide to using the browser-accessible tool based on the model developed in this study


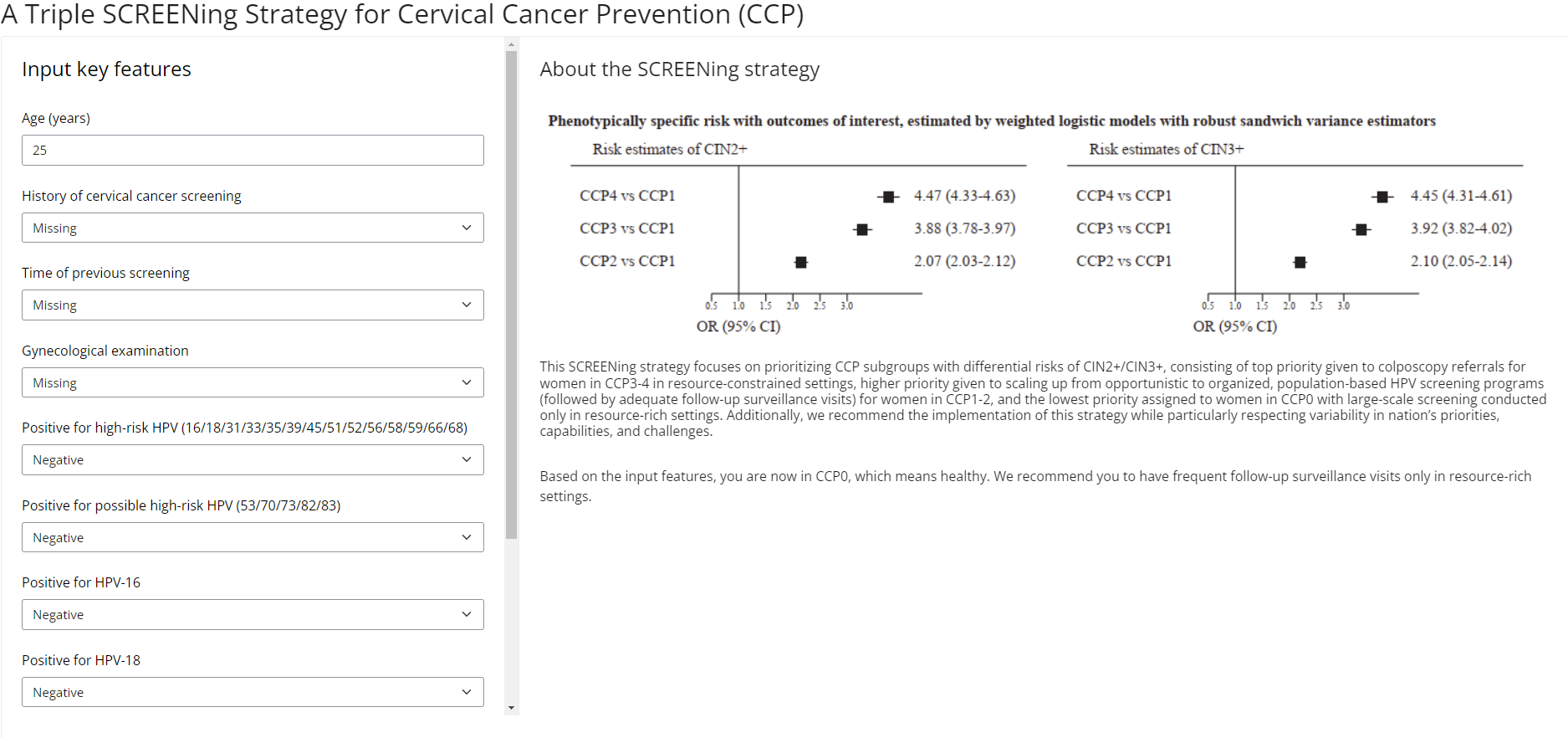


This figure shows a screenshot of our browser-accessible tool based on the model developed in this study, to clarify how the model can be easily accessed via a browser in clinical settings. The model is hosted on a cloud server, and the link https://zhen-lu.shinyapps.io/screening/ can be accessed on mobile phones, iPads, or computers wherever there is network connectivity. Users simply need to click the link, input the required feature values, and they will receive the predicted results from our model. This process is designed to be quick and user-friendly. We believe that this plug-and-play webpage will provide great convenience to our users.

# Reference

1. Bouvard V, Baan R, Straif K, Grosse Y, Secretan B, El Ghissassi F, et al. A review of human carcinogens—Part B: biological agents. The lancet oncology. 2009;10(4):321-2.

2. Cheung LC, Egemen D, Chen X, Katki HA, Demarco M, Wiser AL, et al. 2019 ASCCP risk-based management consensus guidelines: methods for risk estimation, recommended management, and validation. Journal of lower genital tract disease. 2020;24(2):90-101.

3. Schreiberhuber L, Barrett JE, Wang J, Redl E, Herzog C, Vavourakis CD, et al. Cervical cancer screening using DNA methylation triage in a real-world population. Nature Medicine. 2024 2024/06/04. doi: 10.1038/s41591-024-03014-6.

4. Marcus JZ, Cason P, Downs LS, Jr., Einstein MH, Flowers L. The ASCCP Cervical Cancer Screening Task Force Endorsement and Opinion on the American Cancer Society Updated Cervical Cancer Screening Guidelines. Journal of Lower Genital Tract Disease. 2021;25(3).

5. Moy LM, Zhao F-H, Li L-Y, Ma J-F, Zhang Q-M, Chen F, et al. Human papillomavirus testing and cervical cytology in primary screening for cervical cancer among women in rural China: Comparison of sensitivity, specificity, and frequency of referral. International Journal of Cancer. 2010 2010/08/01;127(3):646-56. doi: <https://doi.org/10.1002/ijc.25071>.

6. Saslow D, Solomon D, Lawson HW, Killackey M, Kulasingam SL, Cain J, et al. American Cancer Society, American Society for Colposcopy and Cervical Pathology, and American Society for Clinical Pathology Screening Guidelines for the Prevention and Early Detection of Cervical Cancer. American Journal of Clinical Pathology. 2012;137(4):516-42. doi: 10.1309/AJCPTGD94EVRSJCG.

7. Andersen KM, Bates BA, Rashidi ES, Olex AL, Mannon RB, Patel RC, et al. Long-term use of immunosuppressive medicines and in-hospital COVID-19 outcomes: a retrospective cohort study using data from the National COVID Cohort Collaborative. The Lancet Rheumatology. 2022;4(1):e33-e41. doi: 10.1016/S2665-9913(21)00325-8.

8. Han J, Kamber M, Pei J. Data mining concepts and techniques third edition. University of Illinois at Urbana-Champaign Micheline Kamber Jian Pei Simon Fraser University. 2012.

9. Zheng A, Casari A. Feature engineering for machine learning: principles and techniques for data scientists: " O'Reilly Media, Inc."; 2018. ISBN: 1491953195.

10. Gower JC. A general coefficient of similarity and some of its properties. Biometrics. 1971:857-71.

11. McInnes L, Healy J, Melville J. Umap: Uniform manifold approximation and projection for dimension reduction. arXiv preprint arXiv:180203426. 2018.

12. Oikonomou EK, Spatz ES, Suchard MA, Khera R. Individualising intensive systolic blood pressure reduction in hypertension using computational trial phenomaps and machine learning: a post-hoc analysis of randomised clinical trials. The Lancet Digital Health. 2022;4(11):e796-e805. doi: 10.1016/S2589-7500(22)00170-4.

13. Steinley D. K‐means clustering: a half‐century synthesis. British Journal of Mathematical and Statistical Psychology. 2006;59(1):1-34.

14. Hennig C. Dissolution point and isolation robustness: robustness criteria for general cluster analysis methods. Journal of multivariate analysis. 2008;99(6):1154-76.

15. Reese JT, Blau H, Casiraghi E, Bergquist T, Loomba JJ, Callahan TJ, et al. Generalisable long COVID subtypes: findings from the NIH N3C and RECOVER programmes. eBioMedicine. 2023;87:104413. doi: 10.1016/j.ebiom.2022.104413.

16. Zhou W, He MM, Wang F, Xu RH, Wang F, Zhao Q. Latent class analysis-derived classification improves the cancer-specific death stratification of molecular subtyping in colorectal cancer. NPJ Precis Oncol. 2023 Jun 23;7(1):60. PMID: 37353681. doi: 10.1038/s41698-023-00412-w.

17. Lanza ST, Rhoades BL. Latent class analysis: an alternative perspective on subgroup analysis in prevention and treatment. Prevention science. 2013;14(2):157-68.

18. Nylund KL, Asparouhov T, Muthén BO. Deciding on the number of classes in latent class analysis and growth mixture modeling: A Monte Carlo simulation study. Structural equation modeling: A multidisciplinary Journal. 2007;14(4):535-69.

19. Sclove SL. Application of model-selection criteria to some problems in multivariate analysis. Psychometrika. 1987;52:333-43.

20. Hancock GR, Samuelsen KM. Advances in latent variable mixture models: IAP; 2007. ISBN: 1607526344.

21. Lo Y, Mendell NR, Rubin DB. Testing the number of components in a normal mixture. Biometrika. 2001;88(3):767-78.

22. Sinha P, Calfee CS, Delucchi KL. Practitioner’s guide to latent class analysis: methodological considerations and common pitfalls. Crit Care Med. 2021;49(1):e63-e79.

23. Nguena Nguefack HL, Pagé MG, Katz J, Choinière M, Vanasse A, Dorais M, et al. Trajectory modelling techniques useful to epidemiological research: a comparative narrative review of approaches. Clinical epidemiology. 2020:1205-22.

24. Awada H, Durmaz A, Gurnari C, Kishtagari A, Meggendorfer M, Kerr CM, et al. Machine learning integrates genomic signatures for subclassification b eyond primary and secondary acute myeloid leukemia. Blood. 2021/11/11/;138(19):1885-95. doi: 10.1182/blood.2020010603.

25. Morgan SL, Todd JJ. A diagnostic routine for the detection of consequential heterogeneity of causal effects. Sociological Methodology. 2008;38(1):231-81.

26. Cole SR, Hernán MA. Constructing Inverse Probability Weights for Marginal Structural Models. American Journal of Epidemiology. 2008;168(6):656-64. doi: 10.1093/aje/kwn164.

27. Hernán MA, Robins JM. Causal inference. CRC Boca Raton, FL; 2010.

28. Chen T, Guestrin C, editors. Xgboost: A scalable tree boosting system. Proceedings of the 22nd acm sigkdd international conference on knowledge discovery and data mining; 2016.

29. Zou H, Lu Z, Weng W, Yang L, Yang L, Leng X, et al. Diagnosis of neurosyphilis in HIV-negative patients with syphilis: development, validation, and clinical utility of a suite of machine learning models. eClinicalMedicine. 2023;62:102080. doi: 10.1016/j.eclinm.2023.102080.

30. Al’Aref SJ, Maliakal G, Singh G, van Rosendael AR, Ma X, Xu Z, et al. Machine learning of clinical variables and coronary artery calcium scoring for the prediction of obstructive coronary artery disease on coronary computed tomography angiography: analysis from the CONFIRM registry. European heart journal. 2020;41(3):359-67.

31. Lundberg SM, Erion G, Chen H, DeGrave A, Prutkin JM, Nair B, et al. From Local Explanations to Global Understanding with Explainable AI for Trees. Nat Mach Intell. 2020 Jan;2(1):56-67. PMID: 32607472. doi: 10.1038/s42256-019-0138-9.

32. Bishop C. Pattern recognition and machine learning. Springer google schola. 2006;2:5-43.

33. Jiang LY, Liu XC, Nejatian NP, Nasir-Moin M, Wang D, Abidin A, et al. Health system-scale language models are all-purpose prediction engines. Nature. 2023;619(7969):357-62. doi: 10.1038/s41586-023-06160-y.

34. Collins GS, Reitsma JB, Altman DG, Moons KG. Transparent Reporting of a multivariable prediction model for Individual Prognosis or Diagnosis (TRIPOD): the TRIPOD statement. Ann Intern Med. 2015 Jan 6;162(1):55-63. PMID: 25560714. doi: 10.7326/M14-0697.

35. Lundberg SM, Lee S-I. A unified approach to interpreting model predictions. Advances in neural information processing systems. 2017;30.
